# Technology-enhanced behavior guidance for pediatric dental anxiety: a systematic review and meta-analysis of effectiveness and safety of virtual reality, augmented reality, biofeedback, and games

**DOI:** 10.3389/fdmed.2026.1819864

**Published:** 2026-07-03

**Authors:** Selvakumar Haridoss, Kavitha Swaminathan, Guna Shekhar Madiraju, Ramanathan Ravi, Rohini Mohan, Mamta Thadani

**Affiliations:** 1 Department of Pediatric and Preventive Dentistry, Sri Ramachandra Dental College and Hospital, Sri Ramachandra Institute of Higher Education and Research, Chennai, India; 2 College of Dentistry, King Faisal University, Al Ahsa, Saudi Arabia; 3 Department of Conservative Dentistry, Manipal University College, Melaka, Malaysia; 4 NHS Wales Swansea Bay University Health Board, Port Talbot, United Kingdom; 5 Department of Orthodontics and Paedodontics, University of Ghana Dental School, Accra, Ghana

**Keywords:** augmented reality, biofeedback, digital distraction, gamification, good health and well-being, pediatric dental anxiety, virtual reality

## Abstract

**Background:**

Pediatric dental anxiety is common and can compromise cooperation, increase distress, and contribute to avoidance of dental care. Technology-enhanced behavior guidance (virtual reality, augmented reality, biofeedback, and game-based/digital distraction) is increasingly used, but its effectiveness and safety across pediatric dental procedures remain uncertain.

**Objective:**

To determine whether technology-augmented/digital distraction reduces pediatric dental anxiety/fear and to summarize reported safety/adverse events; secondary objectives were to evaluate effects on pain and physiological arousal (pulse rate).

**Methods:**

This PRISMA 2020–compliant systematic review and meta-analysis was registered in PROSPERO (CRD420251167094). PubMed, Scopus, Web of Science, Google Scholar, EBSCO Dentistry & Oral Sciences Source, and Cochrane Library were searched from inception to 18 December 2025, with additional searches of citation lists, Shodhaganga, and ISRCTN. Eligible English-language studies Evaluated Technology-enhanced/digital Distraction Interventions in Pediatric Dental Care. One Effect Estimate per Study per Outcome was Extracted at an Intra-procedural time Point. Random-effects meta-analyses Were Conducted Using Standardized Mean Differences (SMDs). Risk of Bias was Assessed Using RoB 2 and ROBINS-I, and certainty was rated using GRADE.

**Results:**

Of 675 records identified (590 from databases/registers and 85 from other sources), 61 studies were included qualitatively. For quantitative synthesis, 42 studies contributed to anxiety, 18 to pain, and 30 to pulse rate. Compared with controls, digital distraction reduced anxiety (42 studies; *n* = 2569; SMD −0.97, 95% CI −1.41 to −0.53; I^2^ = 93.8%), reduced pain (18 studies; *n* = 1319; SMD −1.08, 95% CI −1.56 to −0.60; I^2^ = 92.3%), and reduced pulse rate (30 studies; *n* = 1727; SMD −0.61, 95% CI −0.99 to −0.23; I^2^ = 87.9%). Trim-and-fill for anxiety imputed six potentially missing studies and attenuated the pooled effect (adjusted SMD −0.52, 95% CI −1.06 to 0.01). Overall risk of bias was frequently high/with concerns (RoB 2: 25 high, 31 some concerns; ROBINS-I: 4 serious, 1 moderate) and certainty of evidence was very low for all outcomes. Safety reporting was limited; five studies explicitly reported device-related discomfort/adverse effects, with no serious adverse events.

**Conclusion:**

Technology-enhanced behavior guidance may reduce dental anxiety, pain, and physiologic arousal, but very high heterogeneity, frequent risk of bias, limited safety reporting, and sensitivity to small-study effects indicate that the magnitude of benefit is uncertain and likely context-dependent.

**Systematic Review Registration:**

https://www.crd.york.ac.uk/PROSPERO/view/CRD420251167094.

## Introduction

Dental anxiety remains a pervasive challenge in pediatric dentistry and can act as a major barrier to delivering optimal oral healthcare. Dental fear/anxiety in children is commonly conceptualized as an anticipatory emotional response related to dental situations and procedures, and it may contribute to avoidance of dental care and a worsening trajectory of oral health over time (1–3). Among school-age children (7–12 years), prevalence estimates vary by measurement tool and setting, ranging up to 71.3% in girls and 50.4% in boys in one population-based study (4). Anxiety is frequently precipitated by visual and sensory triggers—particularly those linked to injections and operative stimuli; fear of syringes/injections has been directly targeted in clinical studies (5, 6), and the noise of dental drilling is also recognized as a common fear-provoking stimulus in children (7). Clinically, distress may present with physiological arousal (e.g., increased pulse rate), which can complicate behavior guidance and treatment delivery (8). Although pharmacological approaches (including topical anesthetics and, in selected situations, procedural sedation) are used (9), their safety monitoring requirements and feasibility constraints, alongside growing emphasis on conservative behavior guidance, reinforce the importance of effective non-pharmacological strategies to manage anxiety in children (10).

In this context, clinicians have increasingly explored technology-augmented behavioral interventions as digital distraction approaches to reduce anxiety and procedural discomfort, often underpinned by attentional modulation frameworks such as the Gate Control Theory of pain and observational learning principles described in Social Learning Theory (11, 12). Current evidence describes a range of modalities. Virtual reality (VR) aims to immerse children and shift attentional focus away from the procedure, thereby potentially reducing anxiety and pain perception (13, 14). Augmented reality (AR) overlays engaging visual content onto the real environment and has been evaluated during pediatric intra-oral injections with reported benefits on anxiety and pain outcomes (15). Gamified interactive applications similarly aim to enhance engagement and reinforce positive coping behaviors within oral-health or treatment contexts (16). Biofeedback extends this concept by enabling children to observe—and potentially regulate—physiological stress responses during dental care (17). Compared with passive distraction techniques, these technologies are intended to deliver more interactive, multisensory experiences and are typically evaluated against comparator conditions such as usual care or non-digital distraction methods (8, 13, 14).

Despite promising findings from individual trials, the evidence base remains heterogeneous in terms of intervention type, comparators, dental procedures, and outcome measures, limiting confident translation into standardized clinical guidance (8, 13, 16). While existing systematic reviews have synthesized evidence for specific tools (VR) in selected settings (13), uncertainty persists regarding the comparative effectiveness of newer or less-synthesized modalities such as AR, gamification, and biofeedback across different pediatric dental procedures and clinical contexts (16, 17). In addition, reporting of safety and tolerability—such as device discomfort and treatment acceptability—appears variable across studies, complicating risk–benefit interpretation and implementation decisions (13, 15). Therefore, this systematic review with meta-analysis aimed to determine whether digital distraction reduces pediatric dental anxiety/fear and to summarize reported adverse events/safety outcomes. Secondary objectives were to evaluate effects on pain and physiological arousal, including pulse rate.

## Methods

### Protocol registration and reporting

This systematic review and meta-analysis was conducted in accordance with the PRISMA 2020 statement. The review protocol was registered in PROSPERO (CRD420251167094). The final search was completed on 18 December 2025.

### Eligibility criteria

Studies were eligible if they evaluated technology-augmented/digital distraction interventions used during pediatric dental care and reported outcomes relevant to dental anxiety/fear and/or related intra-procedural responses. Digital distraction modalities included immersive or interactive interventions (e.g., VR, AR, gamification/game-based applications, audio distraction, and biofeedback-based approaches). Comparators included usual care, conventional behavior guidance, or non-digital distraction methods.

Primary outcomes were (1) reduction in dental anxiety/fear and (2) safety/adverse events. Secondary outcomes included pain and physiological arousal (e.g., pulse rate). Only English-language studies were included in line with the registered protocol and due to feasibility constraints in ensuring standardized dual-reviewer extraction from non-English publications. Authors were not contacted for missing data.

Both randomized and non-randomized studies were included to capture the full breadth of available evidence. Non-randomized studies were assessed using ROBINS-I and incorporated into the overall synthesis and certainty assessment using GRADE.

Information sources and search strategy. We included only digital distraction interventions delivered during the dental appointment/procedure; pre-visit preparatory or home-based exposure interventions were excluded.

A systematic literature search was conducted from inception to 18 December 2025. Electronic databases included PubMed, Scopus, Web of Science, Google Scholar, EBSCO Dentistry & Oral Sciences Source, and Cochrane. Additional sources included citation searching, Shodhaganga, and ISRCTN. Search strategies were developed and adapted for each database. The full search strategy is provided in Supplementary Appendix 1.

### Study selection

Records were imported into Rayyan for duplicate removal and screening (18). Two reviewers independently screened titles/abstracts and full texts. Disagreements were resolved through discussion, with arbitration by a third reviewer when required. Screening and eligibility decisions were documented using a PRISMA 2020 flow diagram.

### Data extraction

Data were extracted using a standardized Microsoft Excel sheet by two reviewers independently. Extracted variables included study characteristics (year, setting, participant age), intervention/comparator details, procedure type, outcome measures/instruments, and numerical outcome data required for meta-analysis. Discrepancies were resolved by consensus, with arbitration by a third reviewer when necessary.

### Risk of bias assessment

Risk of bias was assessed independently by two reviewers. RoB 2 was used for randomized trials and ROBINS-I for non-randomized studies, where applicable (19, 20). Disagreements were resolved by discussion, with arbitration as needed.

### Outcomes and time-point rule

To avoid non-independence of effect estimates, one effect estimate per study per outcome was extracted for both meta-analysis and publication-bias assessments. Outcomes were preferentially extracted at an intra-procedural time-point (e.g., during local anesthesia administration or during the dental procedure). For studies reporting multiple anxiety measures, we prioritized the most commonly used scale across studies (e.g., Facial Image Scale over less common measures) to facilitate pooling. For physiological outcomes, pre-operative pulse rate values were not extracted. Safety/adverse events were summarized narratively due to variable definitions and inconsistent reporting across studies.

### Data synthesis and statistical analysis

Meta-analysis was conducted using MetaAnalysisOnline (21). Continuous outcomes were synthesized using an inverse-variance random-effects model and reported as standardized mean differences (SMDs) with 95% confidence intervals. Statistical heterogeneity was assessed using the Chi-square test and quantified using the I^2^ statistic (and *τ*^2^ were reported by the software). Prediction intervals were reported when available from the meta-analysis output.

### Publication bias/small-study effects

Small-study effects were assessed using funnel plots and Egger's regression test (21), implemented within MetaAnalysisOnline (22), applying the same unit-of-analysis rule (one effect estimate per study per outcome). Where the platform supported trim-and-fill outputs and asymmetry was suggested, Duval and Tweedie's trim-and-fill method (23) was treated as a sensitivity analysis (not a primary correction).

### Certainty of evidence

Certainty of evidence for key outcomes was assessed using the GRADE approach (24), considering risk of bias, inconsistency, indirectness, imprecision, and publication bias. Summary of Findings tables were prepared for the primary outcome (anxiety/fear) and key secondary outcomes (pain and physiological arousal).

Deviations from Registered Protocol. Minor deviations from the registered protocol (PROSPERO CRD420251167094) are reported in accordance with PRISMA 2020 for transparency. The search strategy was expanded to include Google Scholar and EBSCO Dentistry & Oral Sciences Source to improve sensitivity, and the Cochrane Library was searched as a trial register. Authors were not contacted for missing data due to resource constraints. Egger's regression test and Duval–Tweedie trim-and-fill were added *post hoc* for publication bias assessment given the number of included studies. Prespecified subgroup analyses (age, intervention type, immersion level, and procedure type) were not performed due to insufficient data granularity. Sensitivity analysis excluding high risk-of-bias studies was not feasible, as no study was judged at low risk of bias under RoB 2.

## Results

### Study selection

**Figure 1:**
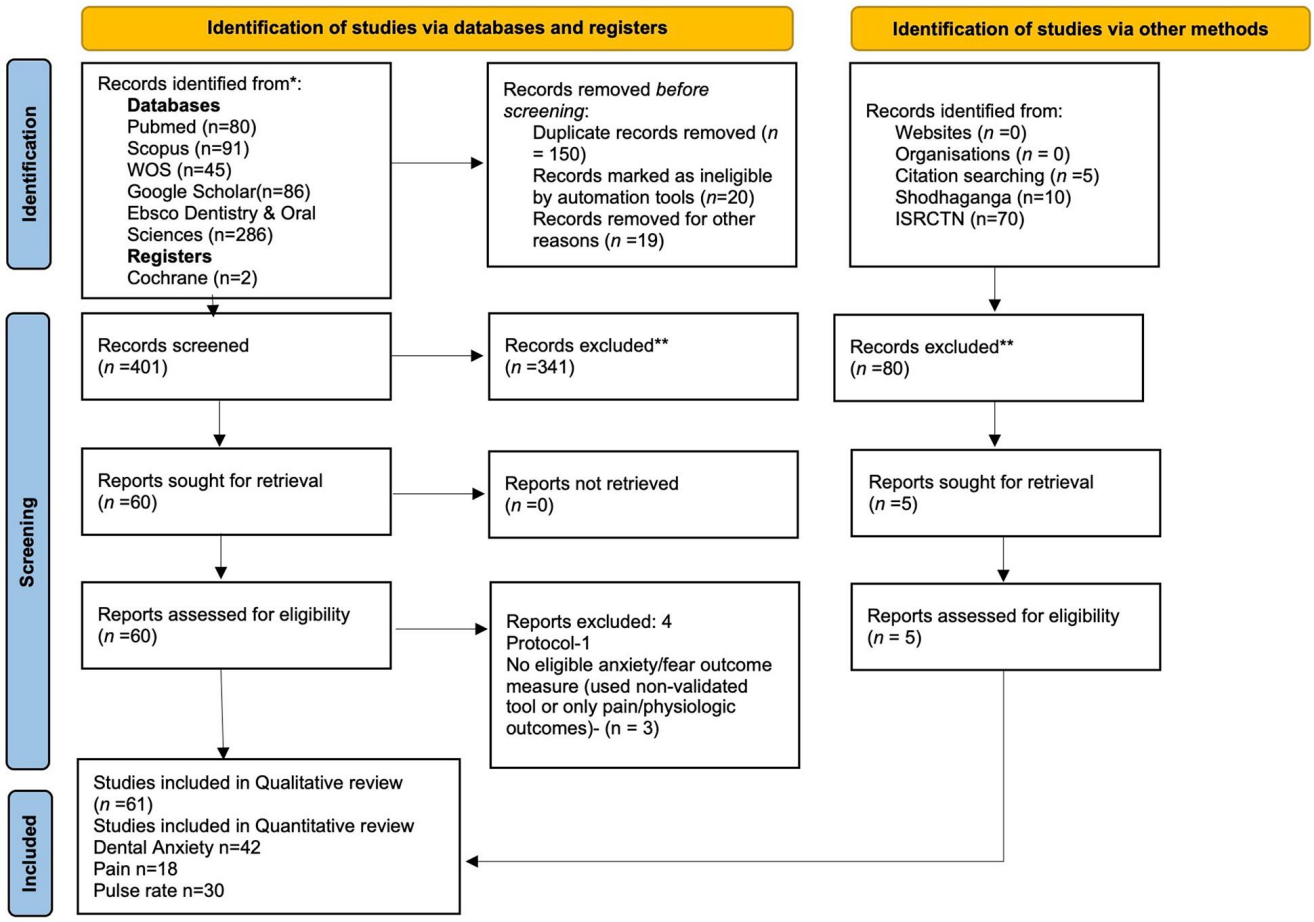
PRISMA 2020 flow diagram for new systematic reviews which included searches of databases, registers and other sources.

The searches identified 590 records from databases and registers (PubMed *n* = 80, Scopus *n* = 91, Web of Science *n* = 45, Google Scholar *n* = 86, EBSCO Dentistry & Oral Sciences *n* = 286, and Cochrane Library *n* = 2), and 85 records from other sources (citation searching *n* = 5, Shodhaganga *n* = 10, and ISRCTN *n* = 70). After removing 189 records before screening (duplicates *n* = 150; records marked as ineligible by automation tools *n* = 20; and records removed for other reasons *n* = 19), 401 records were screened and 341 were excluded at title/abstract screening. Sixty reports from databases/registers and five reports from other sources were assessed for eligibility (total *n* = 65), and four full-text reports were excluded (protocol *n* = 1; No eligible anxiety/fear outcome measure (used non-validated tool or only pain/physiologic outcomes *n* = 3). Details of full-text exclusions and reasons are provided in Supplementary Appendix 2. Overall, 61 studies were included in the qualitative synthesis. For quantitative synthesis, 42 studies contributed data for dental anxiety, 18 for pain, and 30 for pulse rate. The study selection process is summarized in the PRISMA 2020 flow diagram (Figure 1).

### Study characteristics

A total of 61 studies evaluating gamified and/or digital distraction approaches during pediatric dental procedures were included. Key characteristics of the included studies are summarized in (25–85) Supplementary Table S3. Study samples were pediatric populations (predominantly children), with reported ages spanning approximately 4–16 years where stated. Sample sizes varied across studies. Interventions were heterogeneous and included virtual reality–based distraction, digital/mobile or simulation games, and other audiovisual or gamified content, compared against controls such as standard care and/or Tell-Show-Do. Outcomes were assessed using multiple validated anxiety and pain scales, along with physiological parameters (pulse rate), necessitating standardized mean differences for meta-analysis.

### Risk of bias in included studies

**Figure 2:**
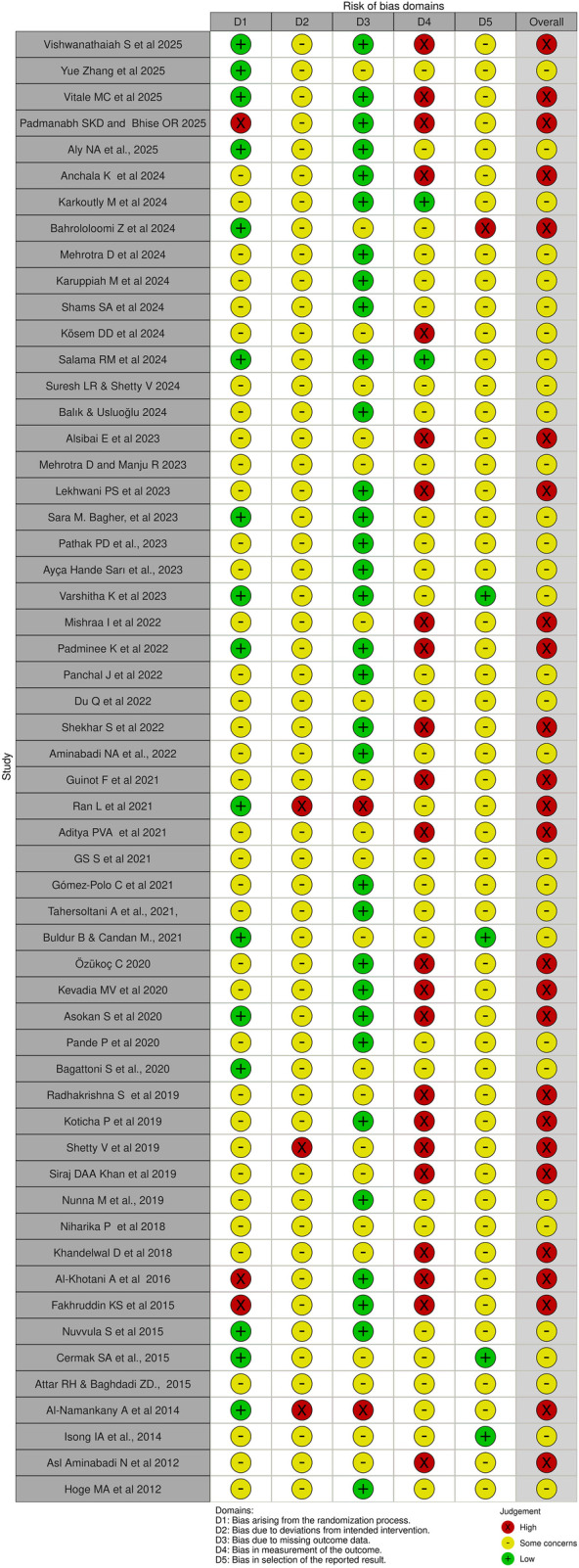
Risk of bias assessment of randomized controlled trials using the RoB 2 tool.

**Figure 3:**
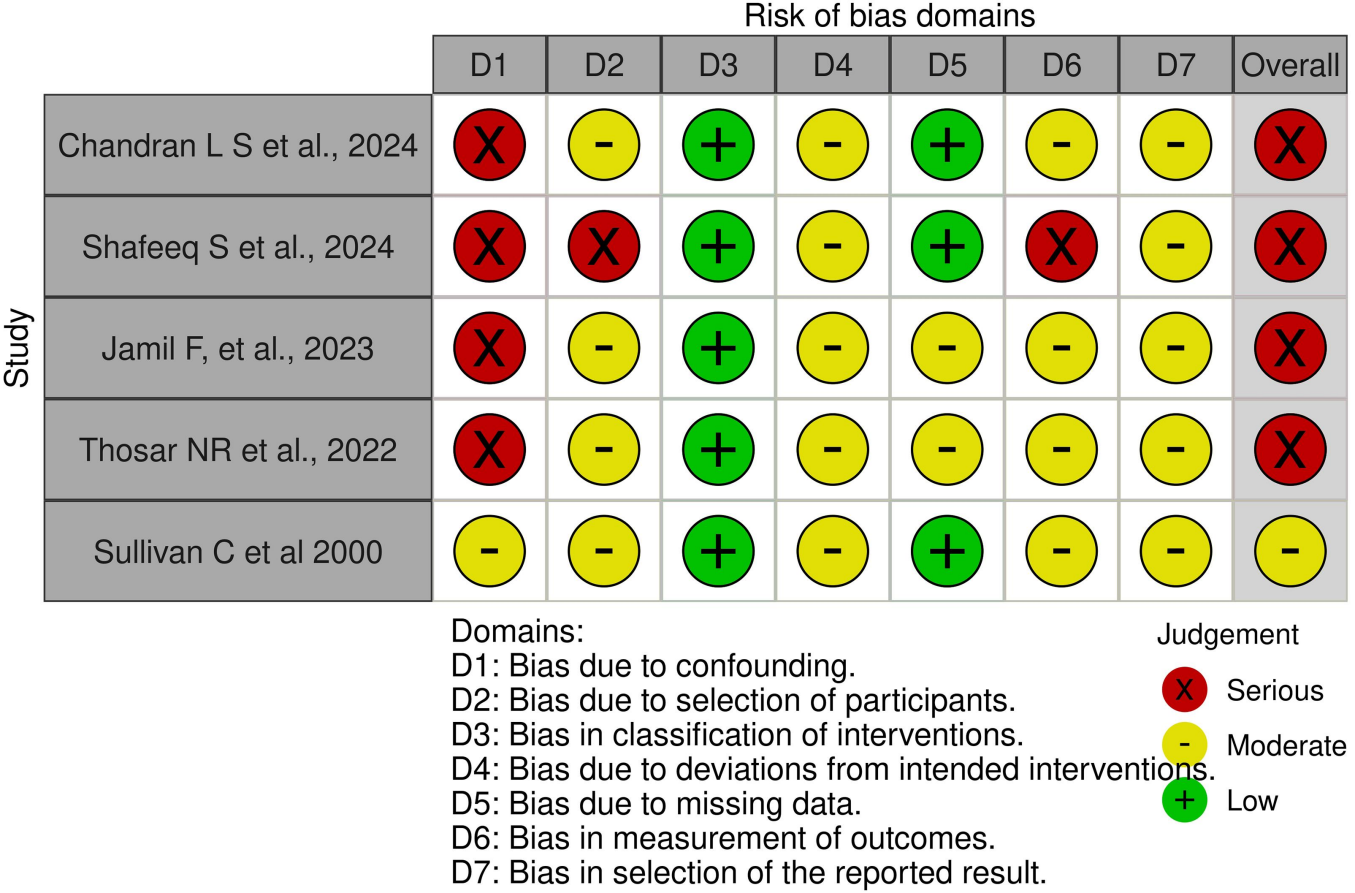
Risk of bias assessment of non-randomized studies using the ROBINS-I tool.

Risk of bias assessments are summarized in Figure 2 (RoB 2) and Figure 3 (ROBINS-I). Among 56 randomized trials assessed using RoB 2, 25 were judged as high risk of bias and 31 as some concerns; no trials were rated low risk overall. Among five non-randomized studies assessed using ROBINS-I, four were judged at serious risk of bias and one at moderate risk.

### Effects of interventions

#### Dental anxiety

**Figure 4:**
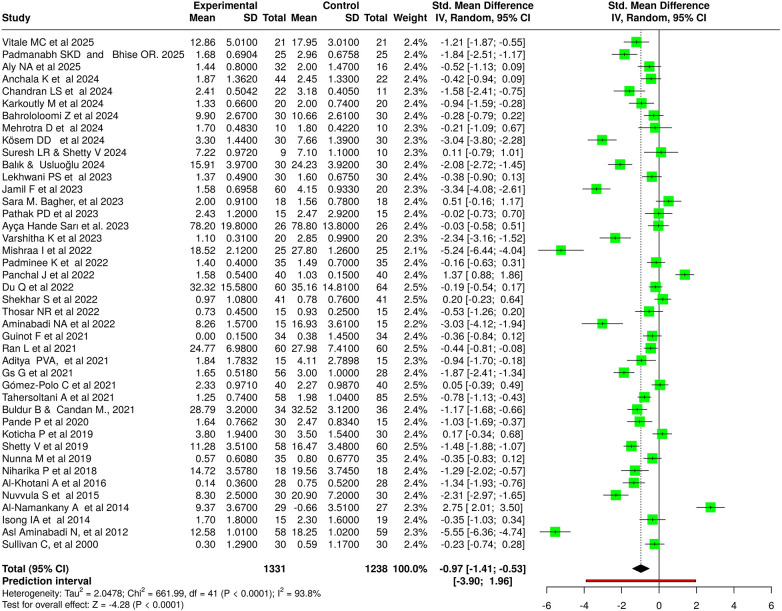
Forest plot showing the pooled effect of technology-enhanced/digital distraction interventions on pediatric dental anxiety.

A random-effects meta-analysis of 42 studies (Experimental *n* = 1331, Control *n* = 1238) showed a statistically significant reduction in dental anxiety with gamified/digital distraction compared with control conditions (SMD = −0.97, 95% CI −1.41 to −0.53). Heterogeneity was considerable (*τ*^2^ = 2.0478; *χ*^2^ = 661.99, df = 41, p < 0.0001; I^2^ = 93.8%). The prediction interval crossed the line of no effect (−3.90 to 1.96), indicating substantial variation in effects across studies. The pooled effects are shown in Figure 4 (anxiety).

#### Pain

**Figure 5:**
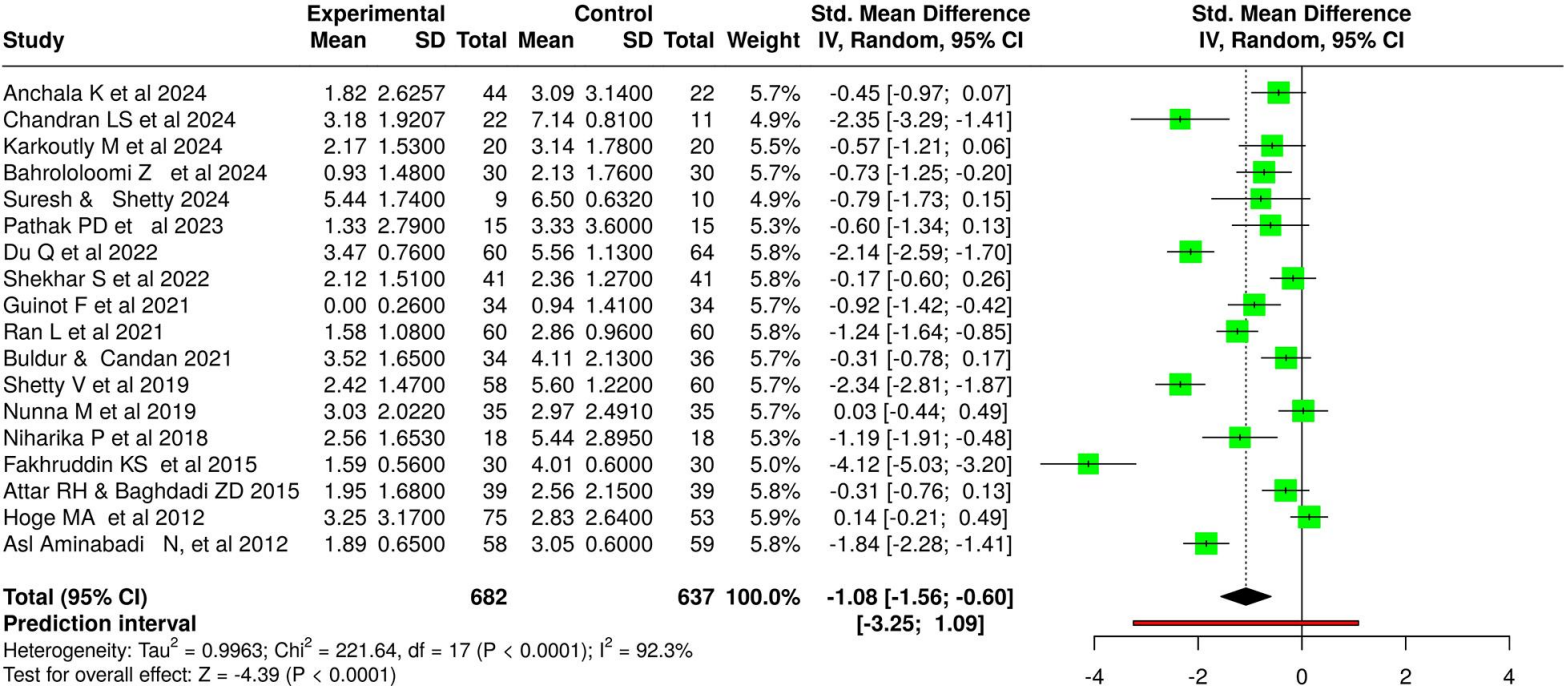
Forest plot showing the pooled effect of technology-enhanced/digital distraction interventions on pain scores during pediatric dental procedures.

A random-effects meta-analysis of 18 studies (Experimental *n* = 682, Control *n* = 637) demonstrated significantly lower pain scores in intervention groups (SMD = −1.08, 95% CI −1.56 to −0.60). Heterogeneity was very high (*τ*^2^ = 0.9963; *χ*^2^ = 221.64, df = 17, *p* < 0.0001; I^2^ = 92.3%), and the prediction interval crossed the null (−3.25 to 1.09). The pooled effects are shown in Figure 5 (pain).

#### Pulse rate

**Figure 6:**
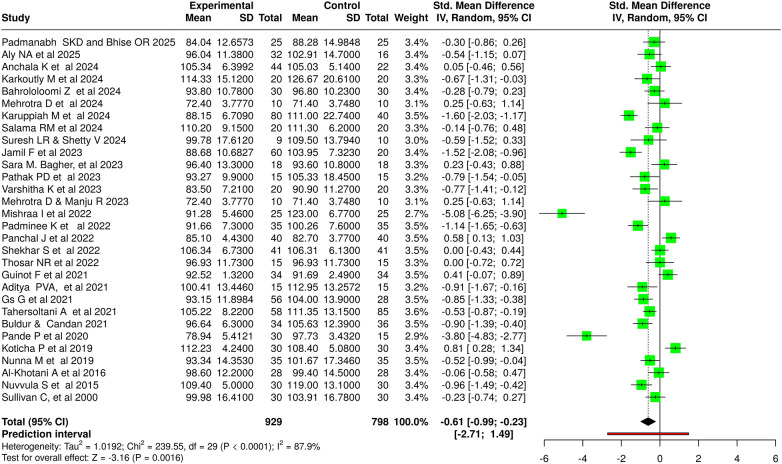
Forest plot showing the pooled effect of technology-enhanced/digital distraction interventions on pulse rate during pediatric dental procedures.

A random-effects meta-analysis of 30 studies (Experimental *n* = 929, Control *n* = 798) found significantly lower pulse rate in intervention groups (SMD = −0.61, 95% CI −0.99 to −0.23). Heterogeneity remained substantial (*τ*^2^ = 1.0192; *χ*^2^ = 239.55, df = 29, *p* < 0.0001; I^2^ = 87.9%), with a prediction interval spanning no effect (−2.71 to 1.49). The pooled effects are shown in Figure 6 (pulse rate).

#### Publication bias

Publication bias/small-study effects were explored using funnel plots and Egger's regression test (Supplementary Figures S1 and S2 for anxiety and pain). In addition, Duval and Tweedie's trim-and-fill method was applied for the primary outcome (anxiety). Six potentially missing studies were imputed, and the adjusted pooled effect was attenuated to SMD −0.52 (95% CI −1.06 to 0.01; random-effects), with substantial residual heterogeneity (I^2^ = 95.4%). Given the very high between-study heterogeneity and variability in interventions and outcome scales, interpretation of funnel plot symmetry, formal tests, and trim-and-fill estimates is cautious. For pulse rate (Supplementary Figure S3), the funnel plot and Egger's regression test were also examined; any apparent asymmetry should be interpreted cautiously because heterogeneity and methodological differences can mimic publication bias.

#### Certainty of evidence (GRADE)

The certainty of evidence was very low for anxiety, pain, and pulse rate (Table 1). Downgrading was primarily due to risk of bias (predominance of high risk/some concerns in RoB 2 and serious risk in ROBINS-I) and very serious inconsistency (I^2^ = 87.9% to 93.8%), with prediction intervals spanning no effect. A separate meta-analysis restricted to non-randomized studies was not performed due to the limited number of eligible non-randomized studies per outcome.

**Table 1:** GRADE summary of findings for technology-enhanced/digital distraction interventions for pediatric dental anxiety (primary outcome), pain, and pulse rate.

Outcome	No. of participants (Experimental/Control)	No. of pooled comparisons*	Effect (random effects, SMD)	Heterogeneity	Certainty of evidence (GRADE)
Anxiety score (lower is better)	2569 (1331/1238)	42	−0.97 (95% CI −1.41 to −0.53)	I^2^ = 93.8%	Very low ⊕○○○^a^^,^^b^
Pain score (lower is better)	1319 (682/637)	18	−1.08 (95% CI −1.56 to −0.60)	I^2^ = 92.3%	Very low ⊕○○○^a^^,^^b^^,c^
Pulse rate (lower is better)	1727 (929/798)	30	−0.61 (95% CI −0.99 to −0.23)	I^2^= 87.9%	Very low ⊕○○○^a^^,^^b^^,d^

* Pooled comparisons = number of effect estimates in the meta-analysis (rows in the forest plot; equals df + 1).

GRADE footnotes.

a (Risk of bias): Downgraded two levels. Most randomized trials had important methodological limitations (RoB2 overall:25 high; 31 some concerns; 0 low). Additionally, included non-randomised studies showed substantial bias (ROBINS-I overall: 4 serious; 1 moderate).

b (Inconsistency): Downgraded two levels due to very high heterogeneity (I^2^ 87.9–93.8%) and variability in effect sizes across studies.

c (Imprecision): Downgraded one level due to wide confidence intervals and variability in pooled estimates across included studies.

d (Publication bias): Downgraded one level due to suspected publication bias and asymmetry observed across included studies.

#### Safety and adverse events

Safety outcomes were infrequently reported across included trials. Five studies explicitly reported safety/discomfort outcomes or adverse effects related to the intervention devices. Du et al. measured simulator sickness using the Simulator Sickness Questionnaire (SSQ) and reported no simulator sickness and no meaningful between-group differences in SSQ scores (53). Bahrololoomi et al. noted headset discomfort related to supine positioning (34), and Mehrotra et al. described paradoxical increases in anxiety/discomfort in some children (35). Mishraa et al. reported lower discomfort in the VR group than comparators (50), and Karuppiah et al. reported low and similar discomfort in VR and 8D audio groups (36). No serious adverse events were reported, but the limited reporting precludes firm conclusions about safety.

## Discussion

This systematic review synthesized contemporary non-pharmacological strategies (digital distraction, VR, gamification, and related approaches) aimed at reducing pediatric dental fear/anxiety and procedural discomfort—problems that are common and clinically consequential because they can drive avoidance and perpetuate a “vicious cycle” of worsening oral health and delayed care (1–3). In current pediatric dentistry guidance, behavior guidance is explicitly framed as a continuum in which distraction and desensitization sit among basic non-pharmacologic techniques, with more advanced options reserved for selected situations (86). This supports the clinical relevance of digital distraction and gamified/immersive tools as pragmatic extensions of established behavior guidance, rather than “add-ons” detached from routine paediatric practice.

Methodologically, the use of standardized mean differences was appropriate because included trials used diverse anxiety and pain instruments and differing procedural contexts; however, the very high heterogeneity and wide prediction intervals emphasize that the pooled estimate represents an average effect across highly variable interventions, populations, and dental procedures. Prediction intervals are particularly helpful in such circumstances because they indicate the plausible range of effects that might be observed in a new setting, and they typically widen substantially as between-study heterogeneity increases (87). We therefore interpreted pooled effects alongside heterogeneity metrics and prediction intervals, and we maintained conservative conclusions consistent with GRADE downgrading for risk of bias and inconsistency (19, 24).

Across outcomes, our findings align directionally with previous pediatric dentistry syntheses, including reviews indicating benefit from VR and other distraction methods in reducing anxiety and/or pain during dental treatment (8, 13). Our results also converge with broader pediatric procedural evidence outside dentistry: meta-analyses have shown that VR distraction reduces pain and anxiety across pediatric medical procedures (e.g., venous access, burns care, perioperative contexts) (88, 89). This cross-context consistency supports the plausibility that immersive or interactive distraction targets shared psychophysiological pathways of anticipatory anxiety and nociceptive salience rather than being procedure-specific.

Mechanistically, digital distraction and immersive VR can be interpreted through attentional models and classic pain modulation concepts. Gate Control Theory proposes that pain perception is not a simple readout of peripheral input but is modulated by competing sensory and cognitive signals (11). Immersive VR strengthens this competition by increasing “presence” (the subjective sense of being in the virtual environment), which has been experimentally linked to larger analgesic effects; likewise, higher display quality and immersiveness can amplify VR analgesia (90, 91). These observations offer a coherent explanatory framework for why more immersive technologies may outperform passive distraction in some trials, while also explaining why benefits can vary widely when immersion, interactivity, and engagement differ across devices and content.

Gamification and interactive games may operate via overlapping but distinct pathways—reward, agency, mastery, and active engagement—potentially improving adherence and cooperation beyond distraction alone (12, 16). Evidence from pediatric procedural care suggests that interactive video games (including VR and non-VR formats) can reduce procedural pain and anxiety (92), reinforcing the biological and behavioral plausibility of game-based distraction during dental injections, restorations, pulp therapy, and extractions. Emerging technologies such as augmented reality and biofeedback may be particularly relevant for anxiety regulation: recent pediatric dental AR trial evidence and biofeedback-based relaxation approaches indicate feasibility and potential benefit, although the evidence base remains smaller and more heterogeneous than VR and audiovisual distraction (15, 17, 51).

In our pooled analyses, digital distraction/gamification approaches were associated with significantly lower anxiety (k = 42) and pain (k = 18), and lower pulse rate (k = 30), suggesting a coherent pattern across subjective and physiologic measures. However, heterogeneity was extreme for all outcomes, and prediction intervals crossed the line of no effect, indicating that some settings may experience large benefit while others may experience minimal or no benefit. Clinically, this implies that “what works” likely depends on context (procedure type, baseline anxiety, novelty/first visit, content appropriateness, interactivity, neurodevelopmental profile, and how the tool is implemented).

### Sources and clinical implications of heterogeneity

Substantial heterogeneity was observed across all outcomes (I^2^ = 93.8% for anxiety, 92.3% for pain, 87.9% for pulse rate), with prediction intervals crossing the null. This reflects variability in intervention type (VR, AR, games, audio-visual distraction, biofeedback), dental procedures, study populations, outcome scales, and comparator conditions. These differences likely contributed to effect size dispersion despite standardization. Clinically, this indicates that pooled estimates should be interpreted as directional effects rather than uniform treatment effects, with effectiveness dependent on context, intervention modality, and procedure type.

### Strength and limitations

This review is strengthened by a large and updated evidence base, inclusion of multiple clinically meaningful outcomes (self-report and physiologic), structured risk-of-bias assessment using RoB 2 and ROBINS-I (19, 20), and incorporation of sensitivity considerations for small-study effects, consistent with best practice for evidence synthesis (24).

First, risk of bias was frequently high or raised concerns, which reduces confidence in effect estimates and justified “very low” certainty ratings (19, 20, 24). Inclusion of both randomized and non-randomized studies may introduce design-related bias; however, non-randomized studies were assessed using ROBINS-I and their influence on certainty was accounted for within the GRADE framework.

Second, interventions and comparators were highly diverse (immersive VR, audio/audiovisual distraction, gamified applications, modeling, and combined techniques), making it difficult to isolate which design elements (immersion, interactivity, content tailoring, or timing) drive benefit.

Third, outcome measures varied substantially, and while SMD allows pooling, it can complicate interpretation and may also interact with small-study effects assessment (93).

Fourth, publication bias and small-study effects should be interpreted cautiously. Egger's regression test is widely used to assess funnel plot asymmetry (21), and trim-and-fill provides a sensitivity approach by imputing potentially missing studies (23). In our updated analysis for anxiety, six studies were imputed and the pooled effect attenuated toward the null (adjusted SMD −0.52, 95% CI −1.06 to 0.01), suggesting potential inflation of effect size. However, funnel plot asymmetry is not equivalent to publication bias, as heterogeneity and other factors may generate similar patterns (94); additionally, SMD-based funnel plots can be inherently distorted, affecting interpretation (93).

Restriction to English-language publications may have introduced language bias by excluding relevant non-English studies.

Taken together, these findings support cautious interpretation: while the direction of effect is consistently favorable, the magnitude of benefit remains uncertain and context-dependent.

Safety reporting was limited and inconsistently captured, restricting conclusions about tolerability. Pediatric dental behavior guidance guidelines emphasize individualized, context-based intervention selection and documentation (86). Future trials should treat adverse events (including device discomfort and simulator sickness) as core outcomes rather than optional reporting.

### Implications for future research

Future research should move beyond simply “VR vs. control” and explicitly test which intervention components matter most: immersion (field of view/audio), interactivity, narrative content, child choice/control, and timing (pre-procedural preparation vs. intra-procedural distraction). Standardization is also needed in outcome measurement: trials should pre-specify primary outcomes, use consistent time points (anticipatory anxiety before injection vs. in-chair anxiety during procedure vs. post-procedure recall), and report both child-reported and observer-rated measures where feasible. Given the limited safety reporting in existing dental trials, adverse effects should be systematically collected using established tools where appropriate (e.g., the Simulator Sickness Questionnaire for VR-related symptoms) (95), alongside documentation of discomfort, withdrawal, and usability barriers. Pragmatic implementation outcomes—setup time, infection-control workflow, staff acceptability, and cost—should be reported to support translation into real pediatric clinics.

### Clinical implications

Given the very low certainty of evidence and substantial heterogeneity, definitive clinical recommendations cannot be made; however, several provisional implications emerge for clinicians considering these interventions. First, digital distraction tools should be viewed as adjuncts to—not replacements for—established behavior guidance techniques (e.g., tell-show-do, positive reinforcement, desensitization, parental involvement), with selection guided by child characteristics (age, developmental status, baseline anxiety, previous dental experience), procedural requirements, and practical feasibility (86). Second, clinicians should closely monitor individual patient response, as benefits may be most pronounced for children with moderate anticipatory anxiety undergoing mildly to moderately invasive procedures (e.g., local anesthesia administration, restorations), while children with severe anxiety, neurodevelopmental disorders, or previous negative experiences may require more intensive or tailored approaches and may not tolerate head-mounted devices (29, 34). Third, systematic documentation of both benefits and adverse effects in routine practice—including whether the child tolerated the intervention, appeared less distressed, or completed treatment more easily—can contribute to practice-based evidence, with validated scales (e.g., Facial Image Scale, Venham) and physiologic monitoring (pulse rate) supplementing clinical judgment where feasible. Fourth, practical considerations require attention, including device fit with child's head size, compatibility with dental procedures (e.g., does the headset interfere with access?), infection control protocols, setup time, and child preference for lighter-weight options (audio-only, tablet-based games) over head-mounted displays when offering choice may enhance acceptability (70). Finally, clinicians should maintain realistic expectations: while pooled effects were statistically significant, prediction intervals crossing the null indicate that some children will experience minimal or no benefit, reinforcing that digital distraction is one tool among many in the behavior guidance armamentarium, not a universal solution.

## Conclusion

This systematic review found that digital distraction and gamification interventions—including immersive VR, interactive games, and related technologies—are associated with statistically significant reductions in dental anxiety, pain, and pulse rate during pediatric dental care. However, the certainty of evidence is very low due to substantial heterogeneity, risk of bias, and concerns about publication bias. Trim-and-fill sensitivity analysis attenuated the pooled anxiety effect toward the null, indicating that the true magnitude of benefit is uncertain and likely context-dependent. These interventions should be viewed as scalable extensions of established behavior guidance rather than replacements for evidence-based techniques. Future research must prioritize high-quality, adequately powered trials with standardized outcomes, rigorous safety monitoring, and clinically meaningful subgroup analyses to determine which children benefit most from which approaches.

## Data Availability

The original contributions presented in the study are included in the article/Supplementary Material, further inquiries can be directed to the corresponding author.
