# Supplementary material for: Technology-enhanced behavior guidance for pediatric dental anxiety: a systematic review and meta-analysis of effectiveness and safety of virtual reality, augmented reality, biofeedback, and games
Source: Front Dent Med. 2026 Jul 3;7:1819864. doi: 10.3389/fdmed.2026.1819864 (PMC13376237; doi:10.3389/fdmed.2026.1819864)
Supplement: Supplementary file 1 [file Table1.docx]

**Supplementary Appendix 1. Search strategy**

Databases searched from inception to 18 December 2025. Language limit: English. No date restrictions.

**PubMed (National Library of Medicine)**

**Search string:**

((("dental anxiety"[tiab] OR "dental fear"[tiab] OR "dental phobia"[tiab] OR "fear of dentist"[tiab] OR "dental distress"[tiab] OR "dental behaviour management problem*"[tiab] OR "dental behavior management problem*"[tiab]) OR ("Dental Anxiety"[Mesh] OR "Fear"[Mesh] OR "Anxiety"[Mesh])) AND (child*[tiab] OR pediatric*[tiab] OR paediatric*[tiab] OR adolescent*[tiab] OR preschool*[tiab] OR "Child"[Mesh] OR "Adolescent"[Mesh] OR "Preschool Child"[Mesh]) AND (dental[tiab] OR dentist*[tiab] OR dentistry[tiab] OR "Pediatric Dentistry"[Mesh] OR "Dental Care for Children"[Mesh]) AND ("virtual reality"[tiab] OR VR[tiab] OR "Virtual Reality"[Mesh] OR "augmented reality"[tiab] OR AR[tiab] OR gamif*[tiab] OR "video game*"[tiab] OR "computer game*"[tiab] OR "mobile game*"[tiab] OR "simulation game*"[tiab] OR "digital distraction"[tiab] OR distraction[tiab] OR "audio distraction"[tiab] OR "audiovisual distraction"[tiab] OR "video eyewear"[tiab] OR "3D video glasses"[tiab] OR "head-mounted display"[tiab] OR biofeedback[tiab] OR "Biofeedback, Psychology"[Mesh])) AND (english[lang])

**Scopus (Elsevier)**

**Search string:**

TITLE-ABS-KEY ( ("dental anxiety" OR "dental fear" OR "dental phobia" OR "fear of dentist" OR "dental distress" OR "dental behaviour management problem*" OR "dental behavior management problem*") AND (child* OR pediatric* OR paediatric* OR adolescent* OR preschool*) AND (dental OR dentist* OR dentistry OR "pediatric dentistry") AND ("virtual reality" OR VR OR "augmented reality" OR AR OR gamif* OR "video game*" OR "computer game*" OR "mobile game*" OR "simulation game*" OR "digital distraction" OR distraction OR "audio distraction" OR "audiovisual distraction" OR "video eyewear" OR "3D video glasses" OR "head mounted display" OR biofeedback) ) AND (LIMIT-TO (LANGUAGE, "English"))

**Web of Science Core Collection (Clarivate)**

**Search string:**

TS=( ("dental anxiety" OR "dental fear" OR "dental phobia" OR "fear of dentist" OR "dental distress" OR "dental behaviour management problem*" OR "dental behavior management problem*") AND (child* OR pediatric* OR paediatric* OR adolescent* OR preschool*) AND (dental OR dentist* OR dentistry OR "pediatric dentistry") AND ("virtual reality" OR VR OR "augmented reality" OR AR OR gamif* OR "video game*" OR "computer game*" OR "mobile game*" OR "simulation game*" OR "digital distraction" OR distraction OR "audio distraction" OR "audiovisual distraction" OR "video eyewear" OR "3D video glasses" OR "head mounted display" OR biofeedback) )

**EBSCO Dentistry & Oral Sciences Source (EBSCOhost)**

**Search string:**

((TI OR AB) ("dental anxiety" OR "dental fear" OR "dental phobia" OR "fear of dentist" OR "dental distress" OR "dental behaviour management problem*" OR "dental behavior management problem*")) AND ((TI OR AB) (child* OR pediatric* OR paediatric* OR adolescent* OR preschool*)) AND ((TI OR AB) (dental OR dentist* OR dentistry OR "pediatric dentistry")) AND ((TI OR AB) ("virtual reality" OR VR OR "augmented reality" OR AR OR gamif* OR "video game*" OR "computer game*" OR "mobile game*" OR "simulation game*" OR "digital distraction" OR distraction OR "audio distraction" OR "audiovisual distraction" OR "video eyewear" OR "3D video glasses" OR "head mounted display" OR biofeedback))

**Cochrane Library (Wiley (CENTRAL))**

**Search string:**

("dental anxiety" OR "dental fear") AND (child* OR pediatric* OR paediatric* OR adolescent* OR preschool*) AND ("virtual reality" OR VR OR "augmented reality" OR AR OR gamif* OR "video game*" OR "computer game*" OR "digital distraction" OR distraction OR "audio distraction" OR "audiovisual distraction" OR biofeedback)

**Google Scholar (web search)**

**Search approach:**

Google Scholar does not support fully reproducible Boolean syntax across time. We used focused keyword combinations and screened the first 200 results (sorted by relevance) for each query. Citations were exported and deduplicated prior to screening.

**Queries used:**

• "dental anxiety" child "virtual reality"

• "dental fear" pediatric "virtual reality"

• "pediatric dentistry" "virtual reality" trial

• "dental anxiety" child gamification OR "video game"

• "dental anxiety" child "digital distraction" OR "audio distraction" OR "audiovisual distraction"

• "dental anxiety" child "augmented reality"

• "dental anxiety" child biofeedback

**Additional sources**

Citation searching (backward/forward), Shodhaganga, and ISRCTN were searched using combinations of keywords: dental anxiety/fear; child/pediatric; virtual reality/VR; augmented reality/AR; gamification/video game/simulation game; digital distraction; audio/audiovisual distraction; biofeedback.
