# Supplementary material for: Technology-enhanced behavior guidance for pediatric dental anxiety: a systematic review and meta-analysis of effectiveness and safety of virtual reality, augmented reality, biofeedback, and games
Source: Front Dent Med. 2026 Jul 3;7:1819864. doi: 10.3389/fdmed.2026.1819864 (PMC13376237; doi:10.3389/fdmed.2026.1819864)
Supplement: Supplementary file 2 [file Table2.docx]

**Supplementary Appendix 2. Full-text studies excluded after eligibility assessment (n = 4)**

This appendix lists reports assessed at full text and excluded, with the primary reason for exclusion, to satisfy PRISMA 2020 item 16b.

| **Excluded study (full citation)** | **Primary reason for exclusion (full text)** |
| --- | --- |
| Parakh H, Thosar N. Evaluation and comparison of the effectiveness of kaleidoscope and virtual reality goggles to reduce dental anxiety in young children undergoing administration of local anesthesia. F1000Research. 2024; 12:546. doi:10.12688/f1000research.134041.4. | Protocol / not a completed trial with extractable outcome data for inclusion. |
| Alsaadoon AM, Sulimany AM, Hamdan HM, Murshid EZ. The Use of a Dental Storybook as a Dental Anxiety Reduction Medium among Pediatric Patients: A Randomized Controlled Clinical Trial. Children (Basel). 2022; 9:328. doi:10.3390/children9030328. | Not a technology-augmented/digital distraction intervention during dental treatment (storybook/educational aid; outside digital distraction scope). |
| Bagher SM, Felemban OM, Alsabbagh GA, Aljuaid NA. The Effect of Using a Camouflaged Dental Syringe on Children’s Anxiety and Behavioral Pain. Cureus. 2023;15(12):e50023. doi:10.7759/cureus.50023. | Not a digital distraction intervention (device camouflage/modified syringe rather than digital/technology-augmented distraction or gamification). |
| Gala UP, Kalaskar R. Comparative Evaluation of the Effectiveness of Innovative Periorbital Eye Massager and  Virtual Reality Eyeglasses for Reducing Dental Anxiety during Dental Restorative Procedures in Children. Int J Clin Pediatr Dent 2024;17(1):48–53. | Not a validated questionnaire used. |
