# Supplementary material for: Technology-enhanced behavior guidance for pediatric dental anxiety: a systematic review and meta-analysis of effectiveness and safety of virtual reality, augmented reality, biofeedback, and games
Source: Front Dent Med. 2026 Jul 3;7:1819864. doi: 10.3389/fdmed.2026.1819864 (PMC13376237; doi:10.3389/fdmed.2026.1819864)
Supplement: Supplementary file 3 [file Table3.pdf]

Supplementary Table 3. Characteristics of included studies evaluating technology-enhanced/digital distraction interventions for pediatric dental anxiety and related outcomes.

S.no	Author, Year, Country	Sample size & Population	Intervention	Control	Outcomes (scales)	Study design	Key findings
1.	Vishwanathaiah S et al., 2025, Saudi Arabia [25]	n=39 children aged 6–10 years each participant acted as own control	Buzzy Bee (cold + vibration) during intra-oral LA administration	Conventional LA infiltration (standard method)	Pain perception: Wong-Baker Faces Rating Scale (WB-FRS/FBRS) (subjective); objective pain response including pulse rate (tool NR)	Split-mouth crossover trial	Buzzy Bee reduced pain perception and increased pain tolerance, especially at the first visit; no statistically significant difference reported at the subsequent visit
2.	Yue Zhang et al (2025), China [26]	n=255**;** 4–6 years children with initial dental fear**,** scheduled for single deep-caries tooth treatment under local anesthesia	Oral Simulation Games	Tell Show Do (TSD)	Child Fear Survey Schedule-Dental Subscale (CFSS-DS), while pain and anxiety levels were measured using the Wong-Baker FACES Pain Rating Scale and Venham's Scale.	Randomized Control trial (RCT)	Significant improvements were observed in pain reduction, fear alleviation, and enhanced compliance in children utilizing oral simulation games
3.	Vitale MC et al 2025, Italy [27]	n=42; 5–10 y; high dental	AI personalised cartoon/anim	TSD	Dental anxiety: MDAS; non-verbal response: FLACC; oral hygiene: OHI-	RCT	Both groups improved from T0→T1 (14 days) with lower anxiety/non-verbal responses and better oral hygiene ($p < 0.05$). Trial group

		anxiety at baseline	ated video + TSD		S, BoP; caries status: ICDAS (T0		showed lower MDAS vs control; regression showed group effect with lower MDAS in trial ($p < 0.05$).
4.	Padmanabh SKD and Bhise OR. 2025,India [28]	n=75; 6–12 y; healthy children; allocated into 3 groups (25 each)	Hand-held video gaming (HHVG); Bubble breath play therapy (BBPT)	TSD	Anxiety (subjective): Facial Image Scale (FIS); objective sign: pulse rate	RCT	BBPT > HHVG for anxiety reduction. Post-treatment intergroup differences vs TSD: pulse rate II vs I p=0.010, III vs I p=0.028; FIS II vs I p=0.001, III vs I p=0.001. Both HHVG and BBPT better than TSD.
5.	Aly NA et al., 2025, Egypt [29]	n=48; children with ADHD; 16/group; multiple visits	VR glasses distraction; White noise (audio) distraction	Control (basic behavior manageme nt) (details NR)	Self-reported anxiety: Faces in the Series (FIS); Anxiety: Venham's scale; Behavior: Visual Behavior Rating Scale (VBRS); Physio: heart rate	Parallel-arm RCT (3-arm)	VR and white noise improved cooperation/behavior vs control; both reduced self-reported anxiety; white noise reported to yield better physiological parameters vs basic behavior management.
6.	Anchala K et al 2024, India [30]	n=66; 6–9 y; LA administrati on; 3 groups (22/group)	VR (VR box); Kaleidoscope ; Mobile video games	Active comparato rs (kaleidosc ope, video games); no separate "no-distraction " control	Anxiety (objective): heart rate (before/during/after); anxiety (subjective): Raghavendra–Madhuri–Sujata pictorial scale (pre/post); pain (subjective): Wong–Baker Faces Pain Scale	RCT	No intergroup differences in heart rate anxiety measures; no significant difference in pictorial anxiety scale. VR group showed significant reduction in pain vs other groups.

7.	Chandran LS et al 2024, India [31]	n=33; 6–9 y; first dental visit; included SCARED < 25	VR distraction device, mobile phone video games	No distraction / normal clinical environment	Anxiety: Facial Image Scale (FIS) (multiple time points: pre-treatment, after pre-op distraction, during LA, post-treatment); Pain: after LA injection (scale NR); handling/ease: questionnaire	Comparative observational study	VR group had lower anxiety after treatment and lower pain perception vs Groups I & II (p < 0.001). Pain during LA highest in control and lowest in VR. VR group reported easier handling/procedure vs mobile phone group.
8.	Shafeeq S et al 2024, Pakistan [32]	n=64; 5–8 y; OPD pediatric dentistry; 32/group	VRDT (Virtual Reality Distraction Technique)	No VRDT	Anxiety: SCARED; Pain: Wong-Baker FACES Pain Rating Scale	Observational Study	VRDT group reported lower pain (mean 1.89 ± 0.65 vs 3.00 ± 0.81, p < 0.05). No difference in anxiety between groups (p > 0.05). Children appeared more relaxed with VRDT.
9.	Karkoutly M et al 2024, Syrian Arab Republic [33]	n=60; 6–10 y; pulpotomy of primary molars; 30/group	Baby Panda Dental Care" mobile game (play 5 min pre-treatment; root canal therapy module)	TSD	Objective anxiety: pulse rate (t0–t3); subjective anxiety: RMS pictorial scale (t0–t3); pain: FLACC (t2)	Triple-blinded RCT (parallel, active-controlled)	Game group showed lower pulse rate at t1 (p=0.012) and t2 (p=0.015) vs TSD. RMS scores differed at t1 (p<0.001), t2 (p=0.006), t3 (p<0.001). FLACC differed between groups (p=0.033). Authors state the game performed better than TSD, but also state neither technique effectively reduced anxiety/pain overall.
10.	Bahrololoomi Z et al 2024, Iran [34]	n=30; 6–8 y; bilateral mandibular primary molar pulpotomy	VR headset/glasses distraction during pulpotomy	No VR during pulpotomy (crossover to contralater	Anxiety: pulse rate (PR), MCDAS; Pain: Wong–Baker Faces Pain Scale (WBFP) (pre/post)	Split-mouth randomized crossover trial	PR**:** no significant difference. MCDAS lower with VR (p=0.02)**;** WBFP lower with VR (p=0.001)**.** Conclusion: VR reduced pain and anxiety during mandibular pulpotomy.

				al tooth at 2nd visit)			
11.	Mehrotra D et al 2024, India [35]	n=20; 6–14 y; mild intellectual disability (DSM-V); ≥1 carious tooth needing restorative treatment	Audio distraction (music); VR distraction (cross-over between visits, 1 month apart)	Active comparator: audio vs VR (no separate no-distraction control)	Physio: pulse rate, SpO_2; Observational anxiety: Venham's Anxiety Rating Scale (3 time points/visit)	Cross over	Both audio and VR produced ↓pulse, ↑SpO_2, ↓Venham scores (significant $p<0.05$ / highly significant $p<0.01$). No between-group difference (audio vs VR) in either visit ($p>0.05$).
12.	Karuppiah M et al 2024, India [36]	n=120; 4–10 y; first dental visit; 3 equal groups	8D audio analgesia + TSD conditioning; VR box + TSD conditioning	TSD	Anxiety/pain: Chotta Bheem–Chutki (CBC) scale, FLACC; Physio: pulse rate, SpO_2	RCT	Both 8D audio and VR showed better outcomes than TSD. No significant difference between 8D audio vs VR (ANOVA + Tukey).
13.	Shams SA et al 2024, India [37]	n=45; 6–10 y; 3 groups	VR distraction; audio distraction	TSD	Anxiety: Modified Venham's Anxiety Rating Scale (MVARS) (pre/post)	RCT	All groups showed significant pre–post anxiety reduction. VR group had the greatest reduction; authors state all methods reduced anxiety, with VR outperforming the others.
14.	Kösem DD et al 2024 [38]	n=120; 7–10 y; tooth extraction + extirpation under LA; 60/group	VR glasses distraction during procedure	Routine care / no VR	Fear: Children's Fear Scale; Anxiety: Child Anxiety Scale–State	RCT	Post-treatment anxiety and fear scores were lower in VR group vs control ($p < 0.001$). Some children requested stopping VR during the procedure (tolerability issue).

15.	Salama RM et al 2024, Egypt [39]	n=40; 5–7 y; moderate–severe hearing impairment; pulpotomy under LA + SSC; 20/group	VR glasses showing sign-language cartoons	Conventional behavior management	Anxiety (physio): heart rate (HR); objective: Venham Clinical Anxiety Scale (VCAS); subjective: modified Facial Affective Scale (FAS)	Parallel-arm RCT	No significant differences between VR vs control for HR/VCAS during LA (p=0.659/0.282), pulpotomy (p=0.482/0.451), SSC prep (p=0.090/0.284). FAS comparable pre/post (p=0.507/0.749). Practical issue: VR size hindered rubber dam placement.
16.	Suresh LR & Shetty V., 2024, India [40]	n=40 recruited; 19 completed [G1=10, G2=9], ASD; 8–15 y; routine non-invasive dental treatment; randomized cross-over	Virtual reality (VR) distraction during treatment	Conventional behavior management (no VR) (cross-over between visits)	Pain: Wong–Baker Faces; Anxiety: Venham Picture Test; Behavior: Frankl Behavior Rating; Physio: salivary cortisol (baseline/pre/post)	Randomized cross-over trial	VR associated with lower pain ratings vs no-VR visit (p=0.012; 0.048); no significant between-visit difference for perceived anxiety/behavior; salivary cortisol increased baseline→pre→post in both conditions.
17.	Balık E & Usluoğlu F., 2024, Turkey [41]	n=60; 7–12 y; first dental treatment; 30/group (block randomization)	VR glasses/games providing pre-treatment information/guidance (VRG)	Control (no VR guidance) (details NR)	Anxiety before treatment; behavioral compliance during treatment (scales NR)	Parallel-arm RCT	VRG reduced pre-treatment anxiety and improved cooperation/compliance during treatment.

18.	Alsibai E et al 2023, Syria [42]	n=105; 6–10 y (mean 7.4); pulpotomy in primary mandibular molars; 35/group	Active distraction (VG): video game on tablet + wireless joystick; Passive distraction (AV): video on tablet + wireless headphones	TSD	Pain: Simplified Wong-Baker FACES (end of treatment); Behavior: HOUPT (end of treatment)	RCT	VG reduced pain vs AV and TSD (p=0.000). VG improved behavior vs TSD (p=0.041); VG vs AV behavior not different (p=0.605).
19.	Mehrotra D and Manju R 2023, India [43]	n=40; 6–14 y; Group I: mild intellectual disability (n=20); Group II: healthy children (n=20); subgroups 10 each; cross-over after 1 month; restorative treatment	Audio distraction (Indian instrumental music) and VR distraction (cross-over)	Active comparator: audio vs VR; comparison across ID vs healthy	Anxiety: pulse rate, SpO_2, Venham's Anxiety Rating Scale (3 time points)	Cross Over	Audio and VR produced ↓pulse, ↑SpO_2, ↓Venham scores in all subgroups. More effective in healthy than mild ID on intergroup comparison. Both techniques considered effective for anxiety reduction during restorative care.

20.	Lekhwani PS et al 2023, India [44]	n=150; 4–8 y; restorative treatment; 5 groups (30/group)	TSD modifications: Tell Play Do (TPD); TPD + smartphone dentist game; Tell Show Play-Doh; Ask-Tell-Ask	TSD	Anxiety: pulse rate, Facial Image Scale (FIS), Venham's Anxiety Scale	RCT	Pulse rate + FIS: anxiety decreased in all groups except Ask-Tell-Ask. Venham: anxiety decreased pre→post in all groups except Tell Play Do and Ask-Tell-Ask. Conclusion: TPD + smartphone game and Tell Show Play-Doh showed encouraging results alongside TSD.
21.	Jamil F et al 2023, India [45]	n=80; 5–8 y; first dental visit; non-invasive restorative treatment	AVD (audiovisual distraction); FM (filmed modeling); AVD+FM	No specific behavior management technique	Physio: pulse rate, SpO_2, body temperature, respiratory rate, salivary cortisol; Anxiety/fear: FIS, FAPS (pre/post)	Experimental in vivo study	AVD+FM was most effective in reducing dental anxiety and fear compared with other groups and control.
22.	Sara M. Bagher, et al., 2023, Saudi Arabia [46]	n=36; anxious children; 6–14 y; prophylactic dental treatment; stratified block randomization	Virtual Reality Distraction (VRD) eyeglasses during prophylaxis	Cartoon video on regular screen	Baseline anxiety: modified Abeer Dental Anxiety Scale–Arabic (M-ACDAS); Anxiety/behavior: Venham Anxiety & Behavioral Rating Scale (VABRS); Physio: salivary cortisol (SCL), heart rate	Parallel-arm RCT	VRD significantly reduced salivary cortisol vs control ($p<0.001$); no statistically significant difference in HR or VABRS between groups.
23.	Pathak PD et al., 2023, India [47]	n=30; healthy children; 6–	VR device during extraction	No VR (standard care)	Anxiety: Venham Picture Test (VPT) + heart rate; Pain:	Parallel-arm RCT	Post-extraction HR increased in control ($p=0.028$) but not in VR group; pre-extraction measures

		12 y; mandibular primary molar extraction; 15/group			Wong–Baker Faces; Behavior: FLACC		comparable; authors conclude VR reduces anxiety during extraction.
24.	Ayça Hande Sarı et al., 2023, Turkey [48]	n=52; 6–11 y; first dental experience; restorative procedures with LA; 26/group	3D VR glasses distraction (cartoons) during procedure	Tell-Show-Do (TSD)	Anxiety: Child Drawing–Hospital Scale (CD:H); CFSS-DS; pulse rate; Pain: Wong–Baker FACES	Parallel-arm RCT	No statistically significant differences between VR and TSD for anxiety (CD:H/CFSS-DS), pulse rate, or pain (p>0.05).
25.	Varshitha K et al 2023, India [49]	n=40; 6–11 y; hearing impairment; oral prophylaxis (noninvasive); 20/group	VR eyewear + modified Tell-Show-Do (MTSD)	MTSD alone	Anxiety: pulse rate (PR) (objective), Facial Image Scale (FIS) (subjective) (pre/post)	RCT	Post-op anxiety improved with VR+MTSD: PR −6.95 vs +8.55 in MTSD (p=0.001). FIS −2.15 vs +0.10 (p=0.033). Conclusion: VR+MTSD effective for anxiety reduction in HI children.
26.	Mishraa I et al 2022, India [50]	n=100; 7–10 y; anxious children needing inferior alveolar nerve block; 4 groups (25/group)	Audio distraction; Audiovisual distraction; VR (3D VR glasses)	LA with no behavior guidance	Anxiety (pre/post): subjective + objective scales (names NR); satisfaction/comfort (NR)	RCT	VR produced the greatest anxiety reduction, followed by audiovisual, then audio (P=0.000). Children reported high comfort/satisfaction with VR; control showed negativism.

27.	Padminee K et al 2022, India [51]	n=70; 7–12 y; LA over 3 visits; 35/group	Biofeedback relaxation (BR) via BrightHearts app (during LA in visits 1–2)	Audio-visual (AV) distraction (during LA in visits 1–2); no intervention for both groups in visit 3	Anxiety: heart rate; Chotta Bheem–Chutki (CBC) scale	RCT	HR higher in AV vs BR during needle penetration and post-intervention ($p < 0.001$). CBC: no significant between-group difference. Conclusion: both effective; BR better on objective HR, subjective similar.
28.	Panchal J et al 2022, India [52]	n=80; 4–7 y; first dental visit; no past dental history; 40/group	Virtual cognitive tool (Roogies app)	Recorded maternal voice via headphones	Anxiety: pulse rate (pre/during/post); Venham Picture Test (VPT)	Comparative study	Maternal voice group showed significant reduction in anxiety (pulse rate and VPT) ($p \leq 0.001$). Virtual cognitive tool also suggested as an anxiety-reducing option.
29.	Du Q et al 2022, China [53]	n=128; children requiring primary tooth extraction under LA	VR helmet during LA + extraction	Traditional behavior guidance	Anxiety: CFSS-DS; Pain: Wong-Baker FACES; Behavior: Houpt scale; Safety: Simulator Sickness Questionnaire (SSQ)	RCT	VR reduced anxiety within-group (CFSS-DS 34.58±6.90 → 32.32±15.58; p=0.02) and reduced pain vs control (WBFS 3.47±0.76 vs 5.56±1.13; p=0.015). No between-group difference in Houpt (p=0.35) or SSQ (p=0.305).
30.	Shekhar S et al 2022, India [54]	n=123; 8–12 y; dental treatment requiring inferior	Stress ball (active distraction) and audio-visual	Basic behaviour guidance without distraction	Anxiety: Modified Child Dental Anxiety Scale (MCDAS) + pulse rate; Behaviour:	RCT (parallel-arm)	No significant between-group differences for anxiety, behaviour, or pain. Within-group anxiety decreased in all groups. Conclusion: stress ball

		alveolar nerve block; 41/group	eyeglasses (passive distraction) during LA administration		Venham's scale; Pain: self-report + observational pain scales		or AV eyeglasses reduced anxiety but not superior to basic guidance.
31.	Thosar NR et al 2022, India [55]	n=30; children 4–11 y; 15/group	Magic tricks (behaviour management aid) used during second visit	Audiovisual aids used during second visit (first visit: no behaviour management for all children)	Hemodynamic parameters: pulse rate, blood pressure, oxygen saturation; Anxiety scales: Venham's Picture Test (VPT) and modified Visual Analog Scale (VAS)	Observational study	Anxiety reduced in both groups after behaviour management. Hemodynamics improved on second visit (↓ pulse/BP, ↑ SpO_2). Both magic tricks + audiovisual aids were effective for reducing dental anxiety
32.	Aminabadi NA et al., 2022, Iran [56]	n=60; healthy children; 4–6 y; 4 groups (15/group)	Immersive VR; semi-immersive VR; non-immersive VR	Tell-Show-Do (TSD)	Anxiety: Modified Child Dental Anxiety Scale–faces (MCDAS[f]); Behavior: Frankl; Oral health: plaque index (follow-up)	Parallel-arm RCT	Immersive VR showed lowest anxiety ($p<.001$) and highest prevalence of positive behavior ($p=.004$) vs other groups; plaque index improved over time with no between-group differences.
33.	Guinot F et al 2021,Spain [57]	n=68 analysed (34+34); children 6–8 y; required ≥2 visits for	Active audiovisual distraction: PlayStation® video games using Rimax®	Passive audiovisual distraction: cartoon film using Rimax® iVision	Parent perception of anxiety: Modified Corah Dental Anxiety Scale; Child anxiety: Venham Picture Test (VPT); Pain: Wong–Baker	RCT	Lower self-reported pain with PlayStation® vs cartoon (significant differences reported). No significant differences for parent-perceived anxiety, child anxiety, global behaviour, or heart rate between active vs passive distraction. HR increased during anesthetic injection

		restorative treatment	iVision 5.0 eyeglasses	5.0 eyeglasses	Faces Scale; Behaviour: Frankl Scale; Heart rate; Preference/satisfaction questionnaire		in each visit. Both methods were well accepted; children wanted to continue using them in subsequent visits.
34.	Ran L et al 2021, China [58]	n=120; children 4–8 y; short-term dental procedures	Virtual reality (VR) distraction during procedure	TSD	Anxiety: CFSS-DS; Pain: Wong–Baker FACES Pain Rating Scale (WBFS/WBFRS); Behavior/Compliance: Frankl Behavior Rating Scale (FBRS) (before & during); Procedure duration (minutes)	RCT	VR produced greater reductions in anxiety vs TSD (median decrease 8 [7–11] vs 5 [5–7], $p<0.05$). Compliance/behavior improved with VR during treatment ($p=0.02$). Pain reduced with VR ($p<0.05$). Shorter procedure time with VR (19.02 ± 5.32 min) vs control (27.80 ± 10.40 min). Some children reported discomfort leading to discontinuation in a few cases .
35.	Aditya PVA et al 2021, India [59]	n=102, enrolled n=60; children 6–9 y requiring IANB for dental treatment; 4 groups (15 each)	Fidget spinner (FS), Kaleidoscope, Virtual Reality (VR) used individually during IANB	No distraction	Anxiety (self-report): Venham Picture Test (VPT); Physiologic: pulse rate and oxygen saturation (SpO_2) via pulse oximeter at before / during / after IAN	Parallel-group RCT	All distraction groups (FS, kaleidoscope, VR) showed significantly lower VPT anxiety scores vs control. FS and VR showed lower mean pulse rate during IANB; kaleidoscope had pulse rate comparable to control. SpO_2 showed no significant intergroup difference ($p>0.05$). Authors conclude FS/kaleidoscope/VR are effective distraction methods for reducing anxiety during IANB.
36.	Gs G et al, 2021, India [60]	n=90; children 6–8 y with moderate	VR distraction during IANB/tooth	TSD	Subjective anxiety: Facial Image Scale (FIS); Physiologic: pulse rate and	Clinical experimental study	Pulse rate decreased significantly post-distraction in all 3 groups ($p<0.01$); lowest post-distraction pulse was in VR. Between-group:

		anxiety (measured by M-DAS/MDAS); required IANB for mandibular tooth extraction; randomly allocated into 3 groups	extraction (plus a separate audio distraction arm as another intervention)		oxygen saturation (SpO_2) by pulse oximeter (pre- and post-distraction)		TSD vs VR pulse difference significant; audio vs TSD (p=0.06) and audio vs VR (p=0.24) not significant. SpO_2 increased significantly in all groups (p<0.01); post-distraction SpO_2 differed between TSD and audio (p<0.05) with higher SpO_2 in audio, while audio vs VR and TSD vs VR were not significant. FIS scores decreased in all groups (p<0.01). Overall relaxation ranking reported: VR > audio > TSD.
37.	Gómez-Polo Cet al 2021, Spain [61]	n=80; children 5–10 y requiring dental treatment over ≥3 appointments; 40/group	VR headset used as distraction during all appointments	No distraction technique (routine care)	Anxiety: Facial Image Scale (FIS) (first vs last appointment); Behaviour: Frankl Behavior Rating Scale (beginning vs end of treatment)	Randomized two arm trail	VR reduced anxiety and improved behaviour versus control: 95% "happy" in VR vs 40% control (anxiety outcome); 100% positive behaviour in VR vs 57.5% control (behaviour outcome).
38.	Tahersoltani A et al., 2021, Iran [62]	n=143; 5–11 y; 58 intervention / 85 comparison; children receiving dental treatment	Dentistry smartphone game (during treatment)	Tell-Show-Do (TSD)	Anxiety: Venham scale; Wong–Baker FACES, Physiologic: heart rate (pulse oximeter)	Randomized clinical trial (coin-flip allocation)	Lower Venham scores with game vs TSD (1.25±0.74 vs 1.98±1.04; p≤0.001) and lower mean HR (105.22±8.22 vs 111.35±13.15; p≤0.001).

39.	Buldur B & Candan M., 2021, Turkey [63]	n=76 enrolled (70 analyzed); 7–11 y; restorative treatment of mandibular first permanent molar; 2-visit crossover	VR distraction during visit	Attention placebo control (protective glasses only)	Objective: heart rate; Subjective anxiety/pain & visit behavior (scales NR)	Randomized within-subject crossover, placebo-controlled trial	VR reduced pain/anxiety vs placebo condition (HR and self-reports); treatment duration similar between conditions and that there were no significant differences in self-reported measures.
40.	Özükoç C 2020,Turkey [64]	n=23 children 10–12 y with MIH-affected teeth undergoing dental treatment	Virtual reality (VR) video games (3D VR games played during treatment)	No VR / routine treatment (children treated without playing VR games)	Children's Perioperative Multidimensional Anxiety Scale (administered before and during treatment)	Experimental study	Children treated while playing VR games were less worried/fearful, less nervous, and thought less about procedure-related pain; compared with no-VR group, anxiety outcomes showed a statistically significant difference ($p < 0.05$).VR video games reduced dental anxiety in children with MIH.
41.	Kevadia MV et al 2020, India [65]	n=75; children 6–9 y; first dental visit; 25/group; study conducted over two visits	Film modeling: visit 1—watched film of dental procedures on child model + oral prophylaxis; visit 2—Class I GIC	Tell-Play-Do (TPD): visit 1—TPD introduced using dental instruments as play objects + oral	Heart rate (HR); Facial Image Scale (FIS); Venham's Pictorial Index (VPI) — recorded before intervention, after intervention, and during procedure at both visits	RCT	TPD showed significantly lower HR, FIS, and VPI compared with film modeling and smartphone app groups (i.e., better fear/anxiety reduction and cooperative behavior). Conclusion: TPD more effective; suggested as a functional alternative to TSD/modeling.

			restoration AND/OR Smartphone dental app: visit 1—used mobile dental application + oral prophylaxis; visit 2—Class I GIC restoration	prophylaxis; visit 2—Class I GIC restoration			
42.	Asokan S et al 2020, India [66]	n=230 children 4–5 y (2 private kindergartens); randomized n=60 children with high anxiety (based on CBC scale); 3 groups	Magic trick distraction during dental visit OR Mobile dental game distraction ("Little Lovely Dentist" app)	TSD	Baseline screening: Chotta Bheem–Chutki (CBC) anxiety scale. Trial outcomes: Readiness to accept dental treatment (time/acceptance) + postoperative anxiety score (CBC)	Double-blinded RCT	Anxiety reduced significantly in all groups (Magic P=0.001; Mobile game P<0.001; TSD P<0.001). Readiness differed between groups (P=0.025); mobile dental game group accepted treatment faster than magic and TSD. Anxiety reduction similar; mobile game superior for readiness.
43.	Pande P et al 2020, India [67]	n=60; children 5–8 y; Frankl negative behavior; requiring	Audio distraction; Audiovisual distraction (AVD) using VR; Mobile	TSD	Physiological: blood pressure, pulse rate. Non-physiological: Facial Image Scale	RCT	Significant post-intervention differences in physiological + FIS parameters across groups; maximum reduction in AVD (VR) group. TSD showed least reduction. AVD (VR) most effective behavior guidance

		restoration; 4 groups (n=15 each)	phone game distraction (each as separate test groups)		(FIS). (Pre- and post-intervention)		technique for reducing dental fear/anxiety in uncooperative children.
44.	Bagattoni S et al., 2020, Italy [68]	n=48; children with Down syndrome needing dental restorations; 24/group	Audiovisual distraction via video eyeglasses	Conventional behavior management	Pain-related behavior: r-FLACC and Frankl; Operator stress: VAS; appointment duration	Parallel-arm RCT	High refusal to wear eyewear (64%); r-FLACC higher (worse) with eyewear vs control (p=0.0155); authors conclude video-eyeglasses distraction not effective in DS.
45.	Radhakrishna S et al 2019, India [69]	n=60; children 4–8 y; Frankl 2 or 3; requiring Class I/II restorations; 3 groups (n=20 each)	Tell-Show-Play-doh (pre-exposure/play-based modeling); Smartphone dentist game (digital pre-exposure/game-based behavior modification)	TSD	Physiological: pulse rate. Subjective: Facial Image Scale (FIS). Behavior: Frankl behavior rating, FLACC. Provider-related: operator compliance questionnaire	RCT	Compared with TSD, both Tell-Show-Play-doh and smartphone game groups showed lower pulse rate, lower FIS & FLACC, higher proportion achieving Frankl 4, and better operator compliance. Both interventions were more effective than TSD for reducing dental anxiety/ improving behavior.
46.	Koticha P et al 2019, India [70]	children 6–10 y; n=30 children with bilateral carious	Virtual reality (VR) eyeglasses (virtual reality audiovisual	Conventional management	Venham's Picture Test (VPT); Physiological: pulse rate, oxygen saturation (SpO_2)	RCT	Physiology improved with VR: intergroup difference in post-extraction pulse rate was statistically significant (p = 0.03). Self-reported anxiety (VPT) did not reduce significantly, so VR improved

		primary molars indicated for extraction; allocated to 2 groups (n=30 each)	system/3D video distraction) during extraction				physiologic indicators but not VPT-based anxiety.
47.	Shetty V et al 2019, India [71]	n=120 children 5–8 y; SCARED < 25; scheduled for short invasive dental procedures; 60/group	Virtual Reality (VR) distraction during procedure	No VR distraction (conventional behavior management/no VR)	State anxiety: revised Modified Child Dental Anxiety Scale (pre & post); Pain: Wong-Baker Faces Pain Rating Scale (end of treatment); Stress biomarker: salivary cortisol (before, during, after)	RCT	VR reduced pain and anxiety: significant reduction in pain perception ($p < 0.001$) and state anxiety ($p = 0.002$). Salivary cortisol reduction was greater with VR ($p < 0.001$), supporting lower physiological stress during treatment.
48.	Siraj DAA Khan et al 2019, India [72]	n=100 children 4–10 y; selected based on negative Frankl behavior ratings; 50/group; scheduled	Passive audiovisual distraction using "Visual Reality Glasses 3D Box" with cartoon clips/VR films shown throughout treatment	no distraction	Dental anxiety: Facial Image Scale (FIS) (pre- and post-operative); Physiological measures: SBP, DBP, pulse rate recorded across visits/treatment phases	Comparative Study	Overall conclusion: passive AV/VR distraction made children less anxious and more cooperative during invasive procedures vs control. Physiology: SBP during LA application in distraction group was significantly higher than control ($p < 0.05$); differences in SBP/DBP/PR otherwise reported as not statistically significant.

		for 3 dental visits					
49.	Nunna M et al., 2019,India [73]	n=70; 7–11 y; LA for pulp therapy/tooth extraction; 35/group; single-blind	Virtual reality (VR) distraction during LA	Counter-stimulation (CS)	Anxiety: pulse rate; Pain: Wong–Baker Faces Pain Rating Scale (WBFPS), VAS; Anxiety/behavior: Venham's Clinical Anxiety Rating Scale (VCARS)	Prospective randomized single-blind parallel trial	VR showed lower VCARS and greater reduction in pulse rate vs CS (p<0.05). WBFPS for needle prick favored CS; overall authors conclude VR more effective for LA-related anxiety/pain.
50.	Niharika P et al 2018,India [74]	n=40 children 4–8 y; required pulp therapy; screened to have no anxiety disorder (SCARED); two sequence groups; 3 consecutive treatment sessions	Virtual reality (VR) eyeglasses used during pulp therapy (crossover: each child received treatment with VR in one session as per randomized sequence)	Pulp therapy without VR (standard care; crossover comparator)	Pain: Wong–Baker FACES Pain Rating Scale (end of session); State anxiety: Faces version of Modified Child Dental Anxiety Scale (MCDAS) (end of session); Physiology: pulse oximeter parameters + heart rate recorded every 10 min	Single-blind randomized crossover controlled trial	VR eyeglasses produced a significant reduction in pain perception (P < 0.001) and state anxiety (P < 0.001) compared with no-VR sessions. Authors conclude VR is a clinically viable, noninvasive adjunct for pediatric pulp therapy; recommend further research.
51.	Khandelwal D et al 2018, India [75]	n = 400 children 5–8 y; first dental visit;	Tell-Show-Do (TSD); Audiovisual	No behavior management	Anxiety measures: Facial Image Scale (FIS), Venham's Picture Test (VPT);	RCT	AVD reduced anxiety more than TSD. TSD + AVD showed an additive effect and was most beneficial for anxiety reduction

		received restorative treatment	distraction (AVD); TSD + AVD (combined)	technique (control)	Physiological: blood pressure, pulse rate, SpO_2; measured at different stages of the visit		during the first dental visit/restorative care.
52.	Al-Khotani A et al 2016, Saudi Arabia [76]	n=56 children 7–9 y (mean 8.2 ± 0.8); children receiving restorative dental treatment over 3 visits	Audiovisual distraction using eyeglass goggle display i-theatre™ showing videotaped cartoons during treatment (AV-group)	No distraction (routine care)	Anxiety/behavior: Facial Image Scale (FIS); Modified Venham's clinical ratings of anxiety & cooperative behavior scale (MVARS); Physiological: blood pressure, pulse rate	RCT	MVARS significantly lower in AV-group vs control (p=0.029); MVARS decreased during treatment in AV-group (p=0.04). Pulse rate increased during LA injection in control (p=0.02) but not in AV-group. Conclusion: AV eyeglass distraction reduces fear/anxiety and improves cooperation during restorative care.
53.	Fakhruddin KS et al 2015, Sharjah, UAE [77]	n=60 children 4–7 y (31 boys, 29 girls); phobic pediatric patients undergoing primary molar pulp therapy over 2	Audiovisual distraction with video eyewear during LA/pulp therapy + computerized delivery system–intrasulcular (CDS-IS) for local	Audiovisual distraction using projector display only (no video eyewear) (in the alternate session; crossover	Anxiety: Modified Child Dental Anxiety Scale—faces (MCDAS(f)) (pre-treatment each visit). Pain: Wong-Baker FACES Pain Scale (post-procedure). Physiological: pulse oximeter readings (incl. heart rate, SpO_2)	Randomized crossover clinical study	Video-eyewear condition associated with lower anxiety scores (group A showed significant pre-op score change across sessions; reported p≈0.03) and marked reduction in self-reported pain when video eyewear used (both groups). AV distraction with video eyewear + CDS-IS improved cooperation.

		sessions (1-week apart); Group A/B: 30 each	anesthetic delivery (crossover: children received video-eyewear condition in one session and no-eyewear condition in the other)	comparator)	recorded every 10 min		
54.	Nuvvula S et al 2015, India [78]	n=90 children 7–10 y (mean 8.4 y); 49 boys, 41 girls; undergoing local analgesia (LA) administration	3D audiovisual distraction using 3D video glasses + basic behaviour guidance	Basic behaviour guidance without distraction. (Also had an “Audio/music” arm: basic guidance + music)	Anxiety (self-report): MCDAS(f). Physiological: pulse rate. Behaviour: Wright’s modification of Frankl behaviour rating scale and Houpt scale. Child preference/satisfaction	RCT (parallel design)	AV (3D glasses) group: highly significant anxiety reduction on MCDAS(f) ($p<0.001$) and improved behaviour on Houpt ($p=0.003$) vs groups. Pulse rate increased significantly in all groups ($p<0.001$) regardless of distraction. 3D video glasses superior to music, with high child satisfaction/preferences for 3D glasses.
55.	Cermak SA et al., 2015, USA [79]	n=44; 6–12 y (22 ASD, 22 typically developing); dental cleaning	Sensory Adapted Dental Environment (SADE)	Regular Dental Environment (RDE)	Pain: Faces Pain Scale–Revised (FPS-R); Sensory discomfort: Dental Sensory Sensitivity Scale (DSSS);	Pilot/feasibility study	SADE showed positive effect sizes for distress/sensory discomfort/pain (per authors) but did not shorten cleaning time (≈5–7 min longer); feasibility supported.

					Physio: electrodermal activity (NS-SCRs)		
56.	Attar RH & Baghdadi ZD., 2015, Saudi Arabia [80]	n=39; mean age 6.27 y; vital pulp therapy; randomized split-mouth	Active distraction: iPad (games/videos)	Passive distraction: audiovisual (AV) glasses	Pain scores (scale NR); behavior scores (scale NR); heart rate	Randomized split-mouth trial	iPad produced lower pain and behavior scores vs AV glasses during LA (p=0.017); children preferred iPad (24 vs 15).
57.	Al-Namankany A et al 2014, London, UK [81]	Children 6–12 y due to receive dental treatment under local anaesthesia (LA). Sample size is inconsistent in the text you shared: it mentions 80 (40/group) and also 180 recruited with exclusions and 68 randomised.	Video modelling (a modelling video shown before LA)	Control video (oral-hygiene instruction video)	Dental anxiety: Abeer Children Dental Anxiety Scale (ACDAS) (pre/post video). Coping during procedure: Visual Analogue Scale (VAS) recorded throughout the subsequent procedure (including at LA administration).	RCT (parallel design)	Video modelling reduced anxiety vs control, with significantly lower anxiety during the dental procedure—especially at LA administration (p<0.001). Practical limitation noted: model child in video may not match every patient's age/gender/ethnicity.

58.	Isong IA et al., 2014, USA [82]	n=80; ASD + dental fear; 7–17 y; two preventive visits 6 months apart; 4 arms	Video peer modeling; video goggles; combination (peer modeling + goggles)	Usual care (control)	Venham Anxiety Score; Venham Behavior Score; pulse rate (pre/post visit)	RCT (pilot)	Groups using video goggles (alone or combined) showed significant decreases in mean anxiety/behavior scores between visits (≈0.9 points; p=.03); no significant change in control or peer-modeling-only group.
59.	Asl Aminabadi N et al 2012, Iran [83]	n=120 healthy children 4–6 y; 60/group; 3 consecutive sessions (1st: fluoride therapy for all; later sessions: restorative treatment with/without VR in crossover).	Virtual reality (VR) eyeglasses used during restorative dental procedures (distraction)	No VR eyeglasses during restorative dental procedures (same child acts as own control in crossover across sessions).	Pain: Wong–Baker FACES Pain Rating Scale. Anxiety: Faces version of Modified Child Dental Anxiety Scale (MCDAS(f)).	Single-blind RCT	Significant reduction in pain perception (P<0.001) and state anxiety (P<0.001) with VR eyeglasses during dental treatment. Conclusion: VR eyeglasses can successfully decrease pain and anxiety in 4–6-year-olds during dental care.
60.	Hoge MA et al 2012, USA [84]	n = 128, children 4–16 years undergoing restorative	Wraparound video eyewear (audiovisual distraction	Sunglasses (typical/standard eyewear control condition;	Observed disruptive behavior during appointment + self-reported pain + patient	RCT	Disruptive behavior was significantly lower with video eyewear: 22.0 ± 22.0 (control) vs 14.9 ± 15.1 (video eyewear); ANCOVA F (1,125) =7.74; p=0.006. Children liked the video

		dental treatment	delivered via eyewear)	no video distraction)	satisfaction/liking of eyewear.		eyewear significantly more than sunglasses
61.	Sullivan C et al 2000, USA [85]	n= 30 healthy children, ages 5 to 7 Y, were in need of dental restorations requiring local anesthesia	Virtual reality (VR) glass at one of the two restorative appointments	Without VR	Frankl Behavior Rating Scale, Koppitz Rating Scale	RCT	No significant differences for anxiety (p = 0.65) or behavior (p = 0.50). pulse rates – Significant difference- virtual reality (X=99.98, SD=16.41) were significantly lower than pulse rates of subject not viewing virtual reality using the glasses (X=103.91, SD=16.78). VR had no significant effect on the behavior or anxiety of the child
