# Supplementary figures and images for: Technology-enhanced behavior guidance for pediatric dental anxiety: a systematic review and meta-analysis of effectiveness and safety of virtual reality, augmented reality, biofeedback, and games
Source: Front Dent Med. 2026 Jul 3;7:1819864. doi: 10.3389/fdmed.2026.1819864 (PMC13376237; doi:10.3389/fdmed.2026.1819864)

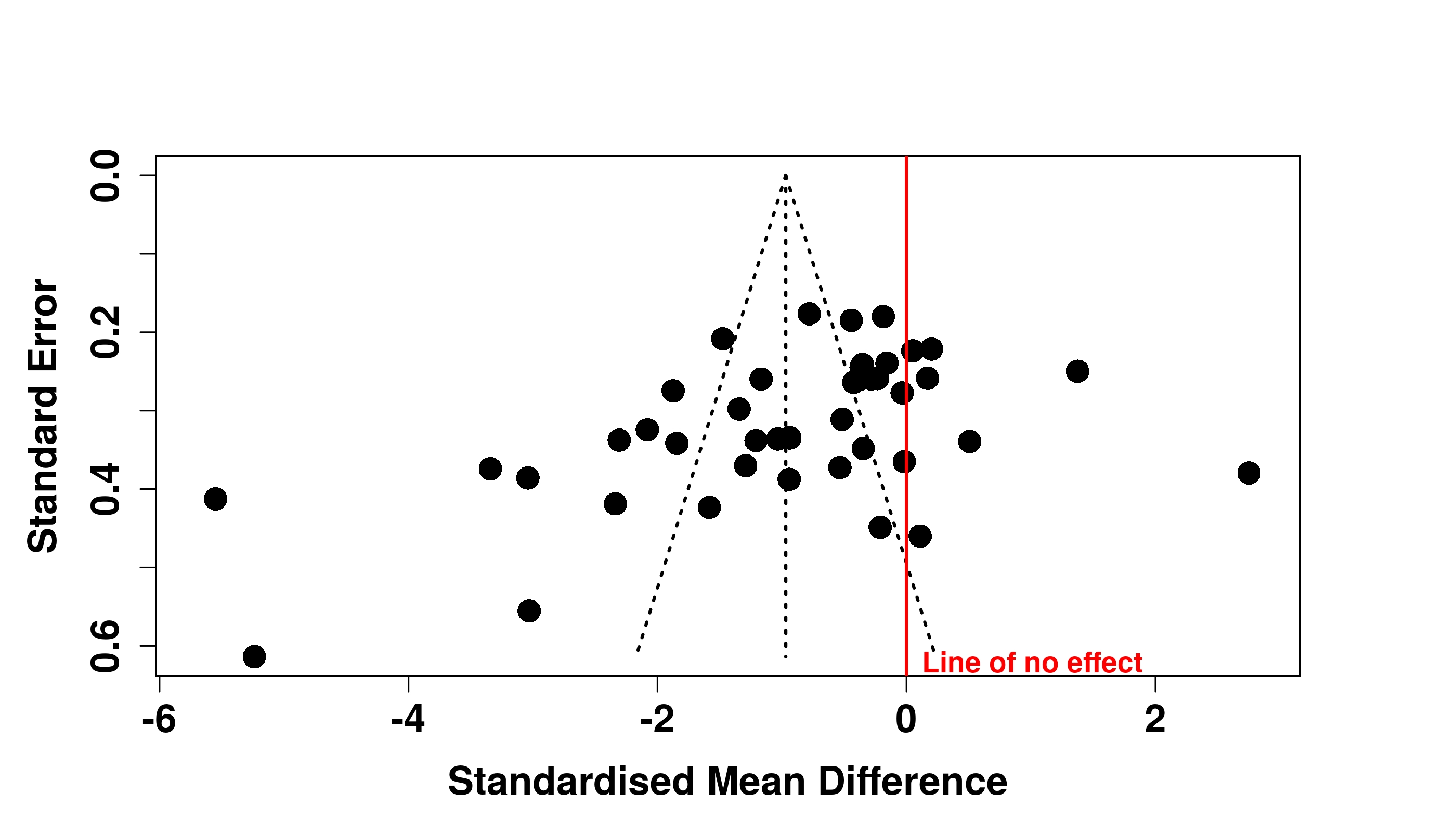

Supplement: Supplementary file 4 [file Image1.jpeg]

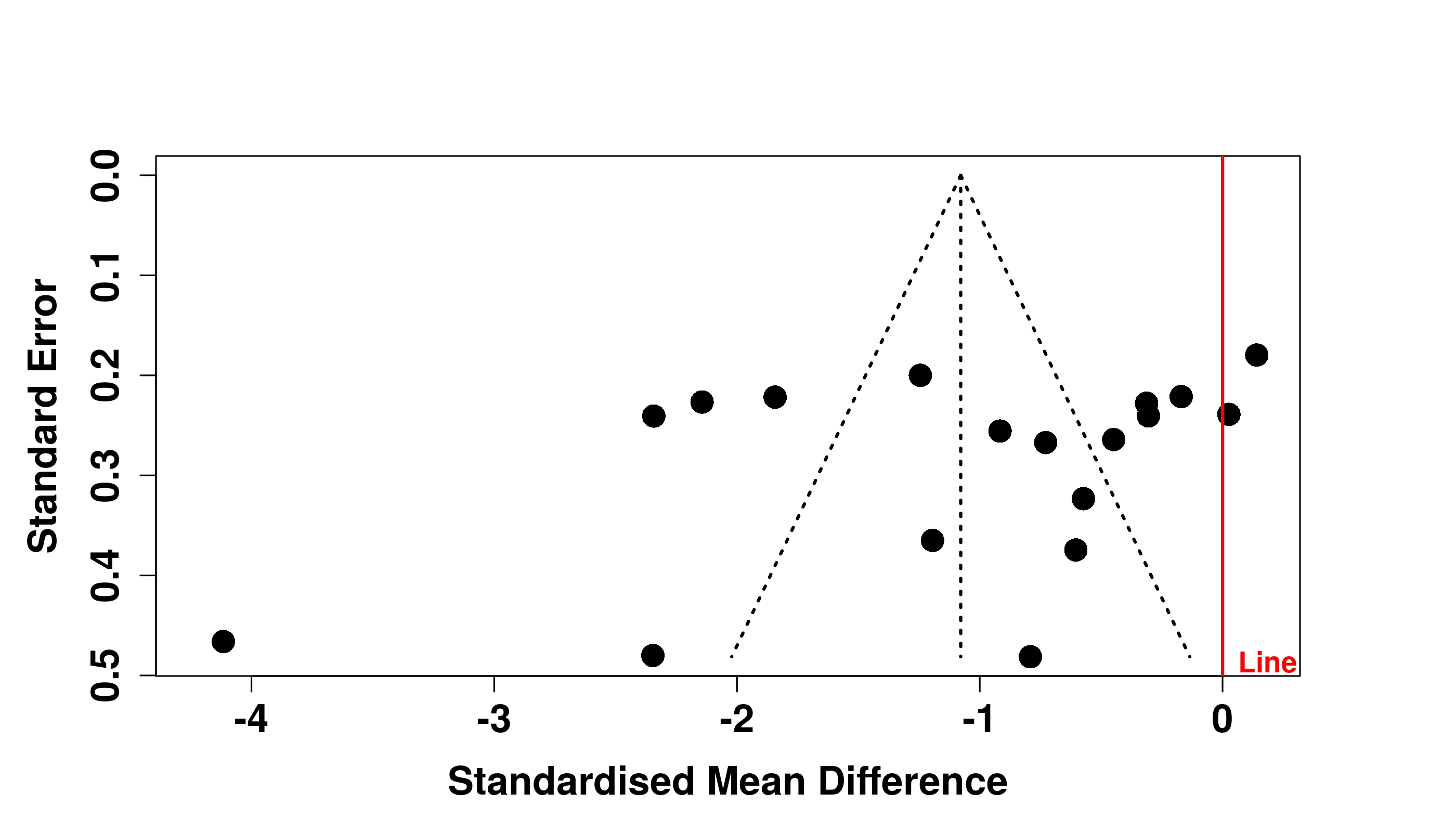

Supplement: Supplementary file 5 [file Image2.jpeg]

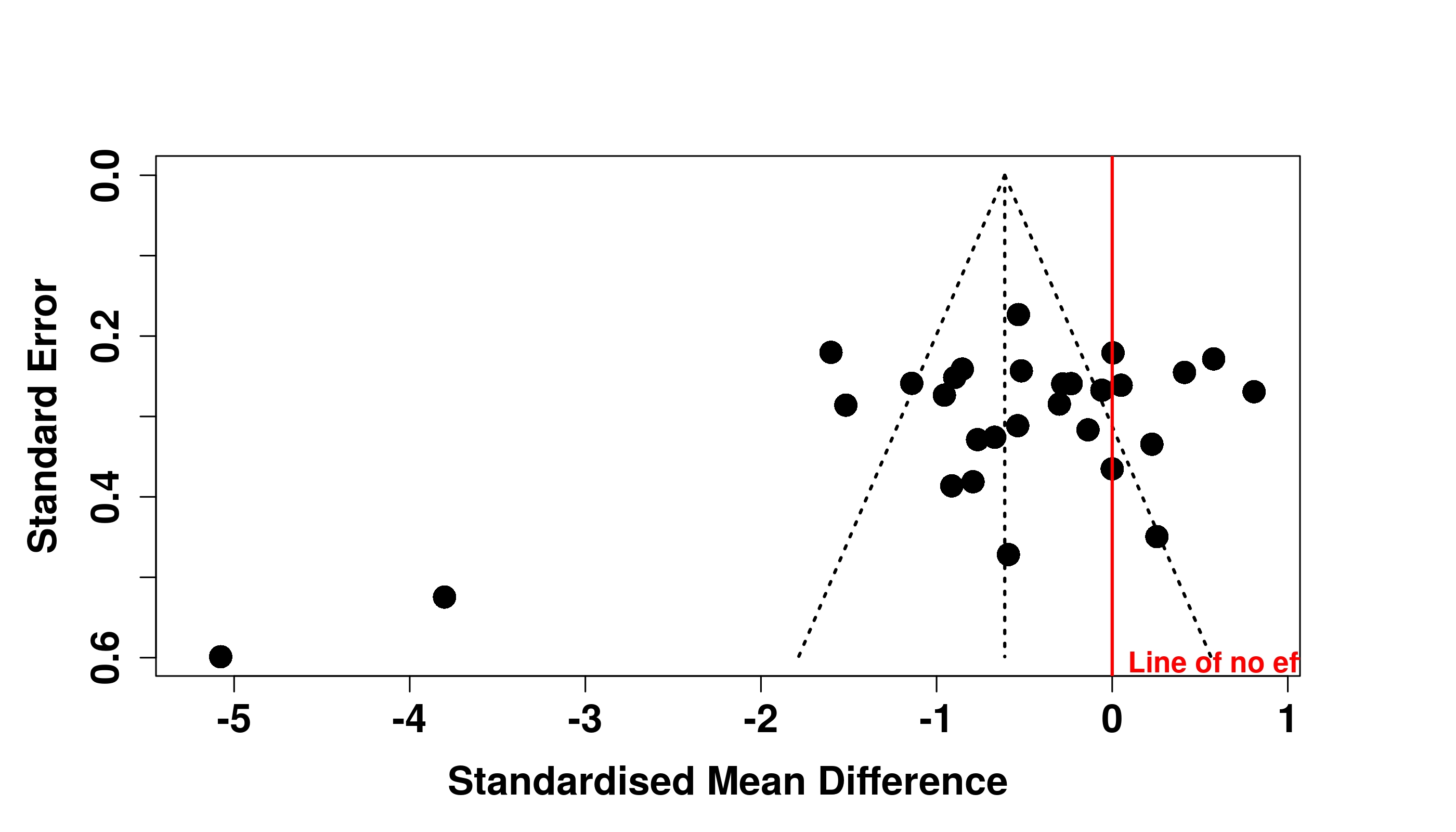

Supplement: Supplementary file 6 [file Image3.jpeg]

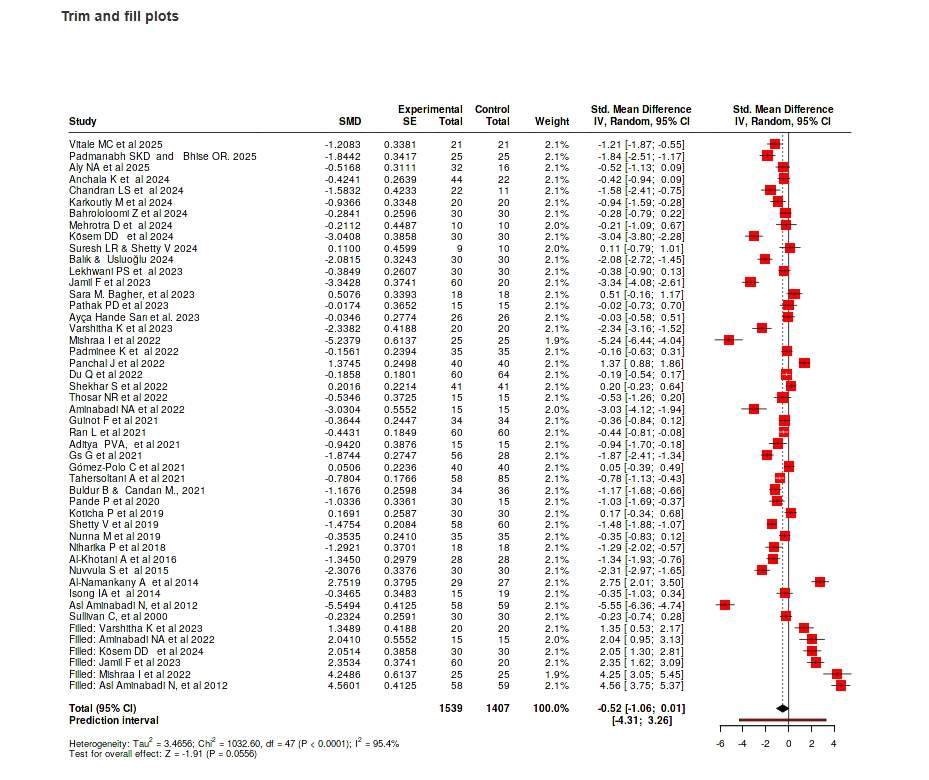

Supplement: Supplementary file 7 [file Image4.jpeg]
